# Autophagy-Modulated Sonodynamic Therapy Triggers Mitochondrial Catastrophe for Potent Immunogenic Tumor Eradication

**DOI:** 10.34133/bmr.0348

**Published:** 2026-04-15

**Authors:** Mengmeng Li, Hua Song, Ya Zhu, Wei Zhang, Tian’an Jiang

**Affiliations:** ^1^ Department of Ultrasound Medicine, The First Affiliated Hospital, College of Medicine, Zhejiang University, Hangzhou 310003 China.; ^2^ Department of General Surgery, Daping Hospital, Third Military Medical University, Chongqing 400042 China.; ^3^ Department of Obstetrics and Gynecology, Chongqing Traditional Chinese Medicine Hospital of Jiulongpo District, Chongqing 400000 China.; ^4^ Department of Ultrasound Medicine, The Second Affiliated Hospital of Chongqing Medical University and Chongqing Key Laboratory of Ultrasound Molecular Imaging, Chongqing 400012 China.

## Abstract

Sonodynamic immunotherapy represents a promising strategy for cervical cancer treatment by stimulating antitumor immune responses. However, therapy-induced prosurvival autophagy may attenuate therapeutic efficacy. To address this limitation, we constructed multifunctional nanoparticles (poly[lactic-*co*-glycolic acid]-b-poly[ethylene glycol] [PLGA-PEG_2,000_]-based nanoparticles coloaded with hematoporphyrin monomethyl ether and SAR405 [PHS NPs]) codelivering the sonosensitizer hematoporphyrin monomethyl ether and the selective vacuolar protein sorting 34 inhibitor SAR405. Upon low-intensity focused ultrasound irradiation, PHS NPs generated reactive oxygen species that induced mitochondrial stress while concurrently modulating autophagic flux through VPS34 inhibition. This coordinated intervention was associated with microtubule-associated protein 1A/1B–light chain 3-II and p62 coaccumulation and the presence of undegraded autolysosomal structures, suggesting impairment of lysosome-associated autophagic degradation. Enhanced oxidative stress, together with modulation of autophagic flux, was accompanied by lysosomal dysfunction and reduced degradative capacity. These alterations were associated with sustained intracellular stress and amplified oxidative injury in tumor cells. Functionally, the combined treatment suppressed tumor growth, promoted immunogenic cell death, and was accompanied by macrophage polarization toward an M1-like phenotype and increased CD8^+^ T cell infiltration. Validated in HPV-associated tumor models, this nanoparticle-based strategy provides a rational and potentially translatable platform to mitigate autophagy-associated adaptive responses and enhance the therapeutic potential of sonodynamic immunotherapy in solid tumors.

## Introduction

Human papillomavirus (HPV) is a leading cause of sexually transmitted infections and a significant factor in the development of cervical, anogenital, and some head and neck squamous cell carcinomas [1]. Among over 200 identified HPV types, HPV-16 and HPV-18 are classified as high-risk strains, responsible for about 70% of cervical cancer cases worldwide [2,3]. Persistent infection with these oncogenic subtypes, especially HPV-16 and HPV-18, is a well-known prerequisite for cervical cancer development [3,4]. Cervical cancer is the fourth most common and deadliest gynecologic cancer globally [5,6]. Although prophylactic HPV vaccines have shown great potential in reducing HPV-related disease burden, vaccine coverage worldwide is uneven, and many individuals are infected before receiving immunization [7,8]. For patients with existing HPV-related cancers, including cervical cancer and head and neck squamous cell carcinoma, standard treatments—such as surgery, chemotherapy, and radiation—often fall short, especially in advanced or recurrent cases [9]. While early-stage tumors can usually be treated successfully with surgery, outcomes for late-stage disease remain poor, highlighting the urgent need for alternative therapies. Therapeutic HPV vaccines offer a promising strategy to eliminate established infections and lesions by generating strong cytotoxic T lymphocyte (CTL) responses against viral antigens persistently expressed in transformed cells [10,11]. The E6 and E7 oncoproteins, which inactivate tumor suppressors p53 and retinoblastoma protein, respectively, promote malignant transformation and are continuously expressed throughout HPV-driven tumor progression [12]. Among these, E7 is considered a particularly promising target for therapy because of its tumor-specific and sustained expression.

However, subunit vaccines composed of purified antigens often exhibit poor immunogenicity and fail to elicit robust cellular immune responses [13,14]. Typically, such antigens are processed via the endosomal–lysosomal pathway and presented by major histocompatibility complex class II (MHC-II) molecules, primarily activating CD4^+^ T helper cells [15,16]. In contrast, CD8^+^ CTL responses—critical for effective tumor elimination—are predominantly induced through MHC-I-restricted antigen presentation [17]. Therefore, strategies capable of enhancing MHC-I-mediated cross-presentation and promoting potent CD8^+^ T cell responses are urgently needed to improve therapeutic efficacy against HPV-related cancers. Modulating intracellular processes such as autophagy, which influences antigen processing and presentation, holds particular promise for augmenting antitumor immunity when combined with physical modalities such as sonodynamic therapy (SDT).

SDT, an emerging biomedical modality that combines low-intensity focused ultrasound (LIFU) with sonosensitizers, offers unique advantages, including deep tissue penetration, spatiotemporal precision, and minimal systemic toxicity, and has been increasingly explored for tumor treatment applications [18–20]. Beyond inducing direct cytotoxicity, SDT has also been shown to promote the release of tumor-associated antigens, thereby potentiating antitumor immune responses [21]. However, the therapeutic efficacy of SDT remains limited. Upon ultrasound activation, sonosensitizers convert molecular oxygen into reactive oxygen species (ROS), primarily singlet oxygen (^1^O_2_), which not only induces oxidative damage but also depletes local oxygen, thereby worsening tumor hypoxia [22–24]. This ROS-induced stress can activate protective mechanisms such as autophagy, ultimately reducing the cytotoxic effects of SDT [25,26]. Furthermore, sustained hypoxia contributes to creating an immunosuppressive tumor microenvironment, which remains a major obstacle to achieving lasting therapeutic responses.

Apart from triggering apoptosis, the release of singlet oxygen (^1^O_2_) during the SDT process may also initiate autophagy [27,28]. Autophagy is a cellular degradation mechanism that involves the breakdown of cytoplasmic proteins or organelles through the formation of autophagic lysosomes. It is widely regarded as a self-protective process aimed at maintaining cellular energy homeostasis and metabolism, as well as facilitating the renewal of damaged organelles [27,29]. However, autophagy acts as a double-edged sword in cancer. While it can suppress tumor formation early on, it can also support tumor cell survival and resistance to therapy during progression [30,31]. Depending on the specific tumor stage [32,33], cell type [34], SDT dosage [35,36], and types of sonosensitizers [37,38], autophagy may either enhance, inhibit, or worsen apoptosis. Importantly, under sublethal SDT conditions (such as low-dose irradiation), autophagy may function as an important resistance mechanism by facilitating the removal of damaged organelles and enabling metabolic adaptation to reduce cytotoxic effects [39]. This protective response may attenuate the effectiveness of SDT in destroying tumors. Therefore, administering autophagy inhibitors along with SDT is a promising strategy to block survival pathways and enhance tumor cell death [26,40]. Although several studies have attempted to enhance SDT by pharmacologically inhibiting autophagy, most approaches have relied on broad-spectrum inhibitors such as chloroquine or 3-methyladenine and primarily demonstrated enhanced cytotoxicity associated with microtubule-associated protein 1A/1B–light chain 3 (LC3) modulation [41,42]. However, these investigations largely interpreted autophagy inhibition at a general level, without systematically dissecting the stage-specific dynamics of autophagic flux under SDT stress. In particular, whether lysosome-associated autophagic degradation and mitochondria-targeted autophagic responses represent critical adaptive mechanisms during SDT remains insufficiently clarified. Therefore, a stage-resolved mechanistic understanding of how autophagy constrains SDT efficacy is critically needed.

Advancing beyond traditional autophagy blockade, SAR405, a selective inhibitor of the class III phosphoinositide 3-kinase vacuolar protein sorting 34 (VPS34), offers dual antitumor benefits by effectively suppressing autophagic flux and reportedly enhancing antitumor immunity through increased infiltration of natural killer cells, CD4^+^ helper T cells, and CD8^+^ cytotoxic lymphocytes [43,44]. This immunomodulatory ability makes SAR405 an attractive candidate for combination strategies aimed at turning immunologically “cold” tumors into responsive microenvironments. Hematoporphyrin monomethyl ether (HMME), a clinically approved porphyrin derivative, has been widely used as an efficient sonosensitizer in SDT because of its strong ROS generating capacity under low-intensity ultrasound. However, its poor solubility, rapid clearance, and limited metabolic stability hinder its therapeutic effectiveness in vivo [45,46]. In addition, suboptimal ROS production may be insufficient to overcome cytoprotective autophagy, a survival mechanism that tumor cells exploit to resist SDT-induced oxidative damage. These challenges highlight the potential of combining SDT with pharmacological inhibition of autophagy. Here, we hypothesize that the efficacy of SDT may be constrained by lysosome-associated autophagic degradation processes involved in the handling of ROS-damaged organelles and stress adaptation. Furthermore, we propose that selective VPS34 inhibition may disrupt this protective autophagic flux at the lysosomal degradation stage, leading to sustained mitochondrial dysfunction, amplified oxidative stress, and enhanced immunogenic signaling.

**Fig. 1.**
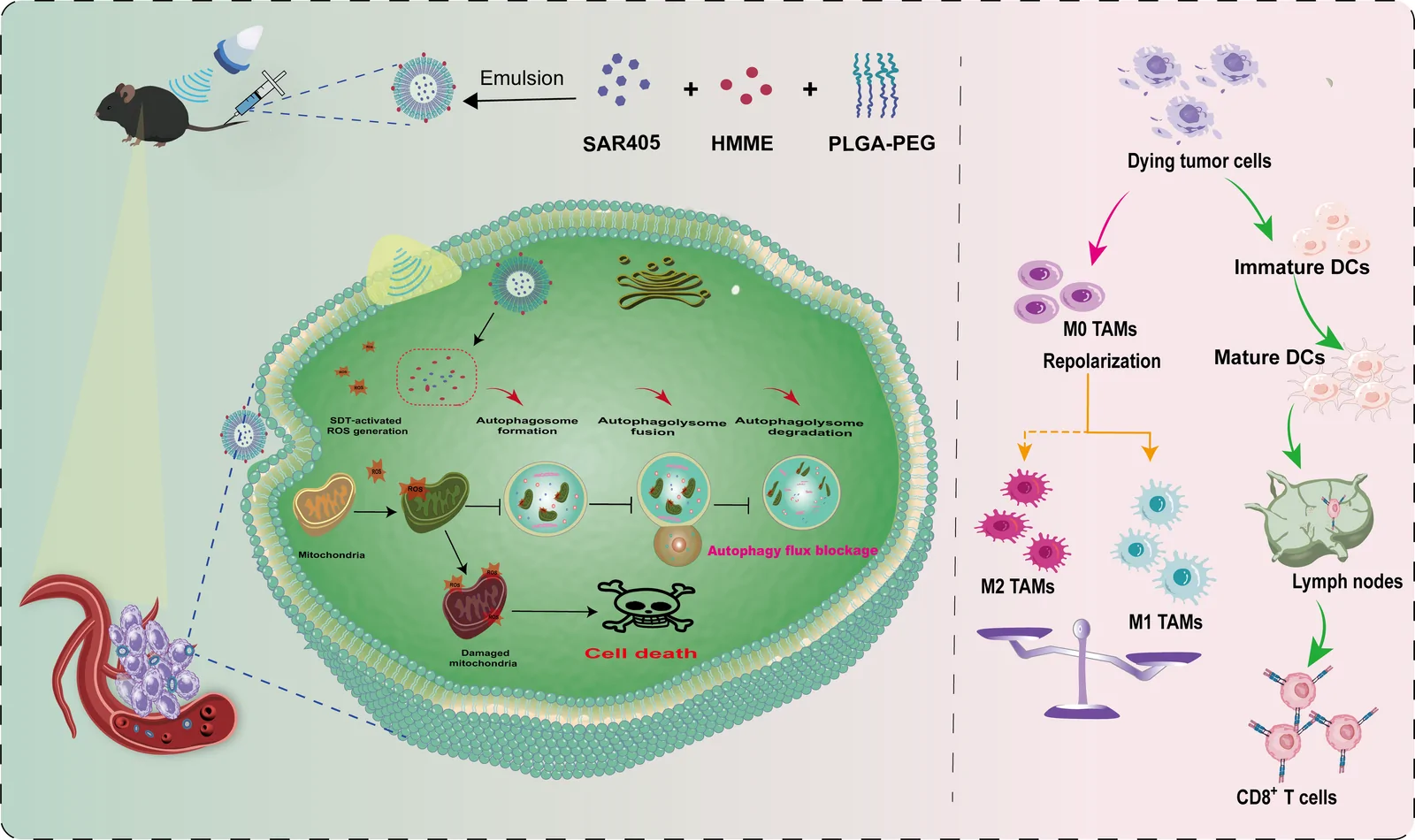
Schematic illustration of the synthesis of poly(lactic-*co*-glycolic acid)-b-poly(ethylene glycol) (PLGA-PEG_2,000_)-based nanoparticles coloaded with hematoporphyrin monomethyl ether (HMME) and SAR405 (PHS NPs) and the ultrasound-activated sonodynamic–autophagy–immune cascade. PHS NPs were formulated by coencapsulating the sonosensitizer HMME and the autophagy inhibitor SAR405 into PLGA-PEG_2,000_. Upon low-intensity focused ultrasound (LIFU) irradiation, the nanoparticles disintegrate, enabling the corelease of HMME and SAR405. HMME produces reactive oxygen species (ROS) under ultrasound-induced sonodynamic activation, while SAR405 interferes with autophagic flux through impaired autolysosomal degradation. The combination of ROS buildup and autophagy inhibition causes lysosomal dysfunction and immunogenic cell death (ICD), thereby triggering antitumor immune activation. TAMs, tumor-associated macrophages.

To test this hypothesis, we developed a functional nanoplatform composed of poly(lactic-*co*-glycolic acid)-b-poly(ethylene glycol) (PLGA-PEG_2,000_) nanoparticles coencapsulating SAR405 and HMME (Fig. 1). In this system, HMME acts as a sonosensitizer, producing ROS upon activation by LIFU, which induces mitochondrial stress and cytotoxic effects in tumor cells. However, increased intracellular ROS may also activate cytoprotective autophagic responses, thereby attenuating the overall therapeutic effectiveness of SDT. To counter this adaptive process, SAR405 selectively inhibits VPS34 and interferes with autophagic flux, thereby limiting engagement of this protective pathway. Importantly, rather than broadly suppressing autophagy, our strategy focuses on functionally perturbing the lysosome-associated stage of autophagic flux, which appears to act as an adaptive regulatory node under SDT-induced stress conditions. By integrating mitochondrial stress with lysosomal degradation dysfunction within a unified framework, this study offers stage-oriented insight into how autophagic flux dynamics may influence SDT responsiveness. Selective modulation of lysosome-associated autophagic processes was associated with sustained cellular stress and enhanced SDT-mediated cytotoxicity. Furthermore, the combined treatment was accompanied by immunogenic cell death (ICD) and modulation of the tumor immune microenvironment, thereby supporting the antitumor immune response.

Overall, these findings highlight stage-resolved characteristics of lysosome-associated autophagic flux under SDT stress and suggest that modulation of this degradation-stage process may represent a rational approach to mitigating adaptive resistance and improving SDT efficacy in HPV-related tumors.

## Materials and Methods

### Materials

PLGA-PEG_2,000_ (lactide:glycolide = 50:50; PLGA molecular weight, 2,000 Da; PEG molecular weight, 2,000 Da) was purchased from Xi’an Ruixi Biotech Co. Ltd. (Xi’an, China). HMME was obtained from Shanghai Yuanye Bio-Technology Co. Ltd. (Shanghai, China), and SAR405 was supplied by GLPBIO Co. Ltd. (Shanghai, China). The ROS assay kit containing the fluorescent probe 2′,7′-dichlorodihydrofluorescein diacetate (DCFH-DA), calcein acetoxymethyl ester (calcein-AM)/propidium iodide (PI) live/dead cell staining kit, 4′,6-diamidino-2-phenylindole (DAPI), JC-1 mitochondrial membrane potential (ΔΨm) detection kit, Cell Counting Kit-8 (CCK-8), The adenosine triphosphate (ATP) Assay Kit and dihydroethidium (DHE) probe were purchased from Beyotime Biotechnology Co. Ltd. (Shanghai, China). Crystal violet staining solution was purchased from Pulilai Co. Ltd. (Beijing, China). LysoSensor Yellow/Blue DND-160 was obtained from Yeasen Biotechnology Co. Ltd. (Shanghai, China). Primary antibodies used in this study included anti-LC3B (rabbit polyclonal), anti-p62/SQSTM1 (sequestosome 1; rabbit polyclonal), and anti-HMGB1 (high-mobility group box 1; rabbit polyclonal), as well as horseradish-peroxidase-conjugated goat anti-rabbit immunoglobulin G (H+L), fluorescein isothiocyanate (FITC)-conjugated, Alexa Fluor 488-conjugated, and Cy3-conjugated secondary antibodies (all from ABclonal Technology Co. Ltd., Wuhan, China, unless otherwise stated). Rabbit anti-calreticulin (CRT) and antilysosomal-associated membrane protein 1 (LAMP1) antibodies were obtained from HuaBio Technology Co. Ltd. (Hangzhou, China). Flow cytometry (FCM) antibodies included fluorophore-conjugated anti-mouse CD80–allophycocyanin (APC), CD86–phycoerythrin (PE), CD206–APC, CD4–FITC, and CD8–PE , CD45-PerCP/Cyanine5.5, CD3-Alexa Fluor 700, CD11c-Alexa Fluor 700, and F4/80-Alexa Fluor 700 (used in separate panels), (all from BioLegend, San Diego, CA, USA). DAPI was added before acquisition to exclude dead cells. Mouse interleukin-6 (IL-6) and tumor necrosis factor-α (TNF-α) enzyme-linked immunosorbent assay (ELISA) kits were obtained from Meimian Industrial Co. Ltd. (Yancheng, China).

### Preparation and characterization of nanoparticles

#### Synthesis of PHS NPs

PLGA-PEG_2,000_-based nanoparticles coloaded with HMME and SAR405 (referred to as PHS NPs) were synthesized using a 2-step emulsion method [47,48]. Briefly, 200 μl of SAR405 solution (10 mM in dimethyl sulfoxide), 5 mg of HMME, and 50 mg of PLGA-PEG_2,000_ were dissolved in 2 ml of dichloromethane and emulsified by probe sonication for 5 min (time on, 5 s; time off, 5 s; power, 35%). Subsequently, 3 ml of a 5% (w/v) polyvinyl alcohol solution was added, and the mixture was further sonicated for an additional 3 min using the same sonication parameters. The resulting emulsion was centrifuged 3 times at 7,000 rpm for 15 min at 4 °C to remove unencapsulated materials and residual solvents. For fluorescence tracing experiments, 1,1′-dioctadecyl-3,3,3′,3′-tetramethylindocarbocyanine perchlorate (DiI)-labeled PHS NPs were prepared following the same procedure with the addition of 1% (w/w) DiI to the organic phase before emulsification. Unless otherwise specified, PH NPs refer to HMME-loaded PLGA-PEG_2,000_-based nanoparticles excluding SAR405, and Blank NPs denote unloaded nanoparticles without either therapeutic agent.

#### Characterization

Nanoparticle dispersions (1 mg/ml in phosphate-buffered saline [PBS]) were vortexed for 30 s and equilibrated at 25 °C for 1 h under standardized ambient lighting before being photographed using a smartphone with fixed exposure settings. The morphological characteristics and structural features of PHS NPs were examined by transmission electron microscopy (TEM; Talos F200X, Thermo Fisher Scientific, USA). The hydrodynamic diameter and zeta potential of PHS NPs were measured using a dynamic light scattering (DLS) analyzer (Zetasizer ZS90, Malvern Instruments, UK). To assess colloidal stability, nanoparticle size in PBS was monitored at predetermined time intervals (days 0, 3, 7, 14, and 21). Ultraviolet–visible–near-infrared (UV–vis–NIR) absorption spectra of HMME were recorded using a spectrophotometer (UV-3600, Shimadzu, Japan). Based on the characteristic absorbance of each drug, the loading capacity (LC) and encapsulation efficiency (EE) of HMME and SAR405 in the nanoparticles were quantitatively determined.The LC and EE were calculated according to the following formulas: LC (%) = (weight of loaded drug / total weight of nanoparticles) × 100, EE (%) = (weight of loaded drug / weight of initially added drug) × 100.

### Cellular experiments

#### Cell culture

TC-1 cells, established initially from C57BL/6 mouse lung epithelial cells cotransformed with the c-Ha-ras oncogene and HPV-16 E6/E7, were obtained from Bluefbio (Shanghai, China). RAW 264.7 murine macrophages and DC2.4 dendritic cells (DCs) were purchased from Procell Life Science & Technology Co. Ltd. (Wuhan, China). TC-1 and RAW 264.7 cells were cultured in Dulbecco’s modified Eagle’s medium supplemented with 10% fetal bovine serum, penicillin (50 U/ml), and streptomycin (50 μg/ml). DC2.4 cells were cultured in RPMI 1640 medium with 10% fetal bovine serum and the same antibiotic concentrations. All cell lines were incubated at 37 °C in a humidified atmosphere with 5% CO_2_.

#### Cellular uptake

The cellular internalization of DiI-labeled PHS NPs was assessed by FCM and confocal laser scanning microscopy (CLSM). TC-1 cells were plated in confocal dishes and allowed to adhere overnight. Cells were then incubated with DiI-labeled PHS NPs (50 μg/ml) for various time points (0.5, 1, 2, 3, and 4 h) under standard conditions (37 °C and 5% CO_2_). After incubation, cells were washed 3 times with PBS to remove uninternalized PHS NPs. For nuclear staining, cells were treated with DAPI (1 μg/ml) for 15 min at room temperature before imaging. Confocal imaging was conducted using a Nikon A1R CLSM system (Nikon, Japan), and quantitative fluorescence intensity was measured by FCM (CytoFLEX LX, Beckman Coulter, USA).

#### Assessment of cell viability and cell death

TC-1 cells were seeded into 96-well plates at a density of 1 × 10^4^ cells per well and allowed to adhere for 12 h. Subsequently, cells were treated with varying concentrations of PHS NPs or free HMME and SAR405 (100 μl per well) for 4, 24, and 48 h. Cell viability was determined using the CCK-8 assay, following the manufacturer’s instructions. Absorbance was measured at 450 nm using a microplate reader (BioTek, USA). For apoptosis analysis, TC-1 cells were seeded in 6-well plates and incubated for 12 h. They were then treated with PHS NPs for 4 h. After incubation, the cells were washed 3 times with PBS, harvested by trypsinization, and resuspended in annexin V binding buffer. Cells were then irradiated with LIFU (2.0 W/cm^2^ for 100 s), incubated for an additional 1 h, and stained with FITC-conjugated annexin V and PI for 15 min at room temperature in the dark. Apoptotic and necrotic cells were analyzed by both CLSM and FCM.

For the clonogenic assay, TC-1 cells were seeded into 6-well plates at a density of 500 cells per well. After adherence, cells were treated according to 5 experimental groups (control, PHS NPs, LIFU, LIFU + PH NPs, and LIFU + PHS NPs). After 7 to 10 d of incubation, visible colonies (≥50 cells) were fixed with methanol and stained with 0.1% crystal violet. Colony images were captured using a smartphone under uniform ambient lighting and consistent camera settings. Colonies were manually counted using ImageJ software.

#### Detection of intracellular ROS

Intracellular ROS levels were measured using the ROS assay kit (DCFH-DA). TC-1 cells were seeded into 6-well plates and cultured for 12 h. The cells were then divided into 5 groups: control, PHS NPs, LIFU, LIFU + PH NPs, and LIFU + PHS NPs. LIFU exposure was applied at 2.0 W/cm^2^ for 100 s in the relevant groups. After treatment, cells were incubated with DCFH-DA working solution for 30 min at 37 °C in the dark. The excess probe was removed by washing with PBS. ROS fluorescence was observed via CLSM and quantified using FCM.

To specifically evaluate superoxide anion (O_2_·^−^) generation, cells were incubated with 5 μM DHE for 30 min at 37 °C in the dark, washed with PBS, and quantified by FCM.

#### Analysis of ΔΨm and ATP levels

ΔΨm was assessed with the JC-1 ΔΨm detection kit (Beyotime, China). TC-1 cells were seeded in 6-well plates and allowed to adhere for 12 h. Following various treatments, cells were washed 3 times with PBS and incubated with JC-1 working solution for 25 min at room temperature in the dark. Mitochondrial depolarization was evaluated by monitoring the fluorescence shift from red (JC-1 aggregates and intact mitochondria) to green (JC-1 monomers and depolarized mitochondria) using CLSM and quantified through FCM. Intracellular ATP levels were also measured after the indicated treatments using an ATP assay kit. Briefly, cells were harvested and lysed, and intracellular ATP content was quantified with a microplate reader. Extracellular ATP levels in culture supernatants were measured using the same assay kit.

#### Maturation of DCs

The TC-1 cells (upper layer) and DC2.4 cells (lower layer) were cocultured for 12 h in a Transwell coculture system (6-well plate and 0.4-μm pore size). After different treatments stimulated the TC-1 cells in the upper layer, the expression levels of DC-specific markers, such as anti-mouse CD80–APC and anti-mouse CD86–PE, were analyzed by FCM. In addition, culture supernatants were collected and analyzed by ELISA to quantify the secretion of TNF-α and IL-6, indicative of DC functional activation.

#### Assessment of macrophage polarization

To assess the impact of various treatments on macrophage polarization, a Transwell-based indirect coculture system was used. TC-1 cells were seeded in the upper chamber of a 6-well Transwell plate (0.4 μm), and RAW 264.7 cells were seeded in the lower chamber. After allowing the TC-1 cells to adhere overnight, they were treated with the indicated formulations and cocultured with RAW 264.7 cells for 12 h under standard incubation conditions (37 °C and 5% CO_2_). Following coculture, RAW 264.7 cells were harvested, washed twice with PBS, and stained with anti-mouse CD86–PE to identify M1-polarized macrophages and anti-mouse CD206–APC for M2 polarization. Samples were analyzed by FCM, and the proportions of CD86^+^ and CD206^+^ populations were quantified.

#### Immunofluorescence staining

TC-1 cells were seeded in confocal dishes at an appropriate density and cultured to 70% to 80% confluency. Immunofluorescence staining for HMGB1, CRT, p62, LAMP1, and LC3B was performed on parallel samples rather than in a single multiplex staining procedure. For mitochondrial–p62 colocalization analysis specifically, cells were preincubated with MitoTracker Red CMXRos (200 nM) for 30 min at 37 °C. All groups then underwent fixation with 4% paraformaldehyde (20 min at room temperature [RT]) and permeabilization with 0.5% Triton X-100 in PBS (20 min at RT). After PBS washing and blocking with 5% bovine serum albumin (BSA) in PBS (1 h at RT), samples were incubated overnight at 4 °C with the corresponding primary antibodies: anti-HMGB1 (1:200), anti-CRT (1:100), anti-p62 (1:200), anti-LAMP1 (1:200) and anti-LC3B (1:500) in 5% BSA/PBS. Following PBS washes, species-matched secondary antibodies (FITC-conjugated anti-rabbit and Cy3-conjugated anti-rabbit; 1 h at RT) were applied, with DAPI nuclear counterstaining (1 μg/ml for 5 min). Confocal imaging was performed using a Zeiss LSM 900 with an Airyscan 2 system, where mitochondrial–p62 colocalization was quantified using ZEN Blue software, while HMGB1, CRT, LAMP1 and LC3B signals were analyzed separately.

For LysoSensor staining, TC-1 cells were incubated with LysoSensor Yellow/Blue DND-160 working solution according to the manufacturer's instructions. After washing with PBS, live-cell images were acquired immediately using a Zeiss LSM 900 confocal microscope. The yellow/blue fluorescence intensity ratio was used to evaluate lysosomal acidification.

#### Western blot analysis

TC-1 cells were seeded into 6-well plates and cultured for 12 h. After various treatments, proteins were extracted using radioimmunoprecipitation assay lysis buffer, which was mixed with a cocktail and phenylmethylsulfonyl fluoride. Sodium dodecyl sulfate–polyacrylamide gel electrophoresis was then used to separate the cell samples, which were transferred to a polyvinylidene difluoride membrane and incubated with fresh blocking solution for 1 h at room temperature. The polyvinylidene difluoride membrane was incubated overnight at 4 °C with primary antibodies anti-p62 (1:1,000) and anti-LC3B (1:500), followed by a 1-h incubation at room temperature with a secondary antibody (1:5,000). Finally, protein bands were visualized using enhanced chemiluminescence and captured with a Bio-Rad ChemiDoc MP imaging system.

### Biosafety evaluation

#### Animal model

Female C57BL/6 mice (6 to 8 weeks old, 16 to 22 g) were purchased from the Laboratory Animal Center of Chongqing Medical University (Chongqing, China). The care and maintenance of the mice followed the protocols established by the Institutional Animal Care and Use Committee of Chongqing Medical University.

#### Biosafety in vivo

The biosafety of PHS NPs was studied in mice. The mice received tail vein injections of PHS NPs at a concentration of 3.63 mg/ml (corresponding to HMME [3 mg/kg]/SAR405 [0.52 mg/kg], 200 μl per mouse). Peripheral blood was collected and analyzed on days 0, 7, 14, and 21. The collected blood was transferred into a 3-ml tube designed to promote coagulation. It was allowed to clot at room temperature for 30 min before centrifugation at 3,500 rpm for 15 min at 4 °C. The serum was collected and analyzed for levels of liver biomarkers (alanine aminotransferase [ALT], aspartate aminotransferase [AST] and total bilirubin), kidney biomarkers (creatinine and blood urea nitrogen [BUN]), and myocardial enzyme spectrum (lactate dehydrogenase [LDH] and creatine kinase [CK]). Three mice were euthanized at each time point, and major organs (heart, liver, spleen, lung, and kidney) were collected, fixed, and stained with hematoxylin and eosin (H&E) for histological examination.

#### Antitumor efficacy in vivo

TC-1 tumor-bearing C57BL/6 mice were randomly assigned to 5 groups (*n* = 5 per group) when tumor volumes reached approximately 100 mm^3^: (a) control, (b) LIFU, (c) PHS NPs, (d) LIFU + PH NPs, and (e) LIFU + PHS NPs. Groups (c) and (e) were intravenously administered PHS NPs at a concentration of 1.28 mg/ml (200 μl per mouse, corresponding to HMME [1 mg/kg] and SAR405 [0.18 mg/kg]), while group (d) received PH NPs at 1.29 mg/ml (200 μl per mouse, HMME [1 mg/kg]). Twenty-four hours after injection, the mice underwent LIFU treatment (2.0 W/cm^2^ for 100 s). Tumor volume (0.5 × length × width^2^) and body weights were monitored every other day throughout the treatment period. Twenty-four hours after the treatment, 5 mice from each group were sacrificed. Subsequently, tumor tissues were harvested and sectioned for terminal deoxynucleotidyl transferase-mediated deoxyuridine triphosphate nick end labeling (TUNEL) assay, H&E staining, and Ki-67 immunohistochemical staining.

#### Antitumor immune activation in vivo

To evaluate immune activation induced by PHS NPs combined with LIFU in TC-1 tumor-bearing mice, single-cell suspensions from spleens and tumors were prepared through enzymatic digestion (Dulbecco’s modified Eagle’s medium containing collagenase [1 mg/ml], hyaluronidase [1 mg/ml], and deoxyribonuclease I [0.5 mg/ml]; 37 °C for 30 min), followed by 40-μm filtration and red blood cell (RBC) lysis. Splenocytes were stained with fluorophore-conjugated anti-mouse CD45, CD3, CD4, and CD8 antibodies for T-cell analysis (helper T cells, CD45^+^CD3^+^CD4^+^; cytotoxic T lymphocytes, CD45^+^CD3^+^CD8^+^). Tumor-infiltrating immune cells were stained with fluorophore-conjugated anti-mouse CD45, CD11c, CD80, and CD86 antibodies for dendritic cell maturation analysis (CD45^+^CD11C^+^CD80^+^CD86^+^) and with anti-mouse CD45, F4/80, CD86, and CD206 antibodies for macrophage polarization analysis (M1 phenotype, CD45^+^F4/80^+^CD86^+^). FCM was performed on a Beckman CytoFLEX LX and analyzed with FlowJo v10. After doublet exclusion and dead cell exclusion, immune cell subsets were analyzed within the CD45^+^ population.

For histology, serial tumor sections underwent independent immunofluorescence staining. The sections were fixed in 4% paraformaldehyde, blocked with 5% BSA, and then incubated overnight at 4 °C with anti-CD8a (1:100). This was followed by incubation with an Alexa Fluor 488-conjugated secondary antibody (1:500) and DAPI. Identical procedures were separately conducted for CD4 staining (anti-CD4, 1:150; Cy3-conjugated secondary, 1:500) and CD86 staining (anti-CD86, 1:200; Alexa Fluor 488-conjugated secondary, 1:500). Imaging was performed using a Zeiss LSM 900 confocal microscope (Carl Zeiss, Germany).

### Statistical analysis

Data were shown as the means ± SD. Significance was tested by one-way analysis of variance (ANOVA) with Bonferroni posttest or Dunnett posttest. For longitudinal tumor growth curves, statistical comparison was performed on endpoint tumor volumes at day 14. Statistical significance is indicated directly in the figure. All analyses were conducted using GraphPad Prism 9.0 (GraphPad Software). A *P* value less than 0.05 was considered statistically significant.

## Results and Discussion

### Preparation and characterization of PHS NPs

**Fig. 2.**
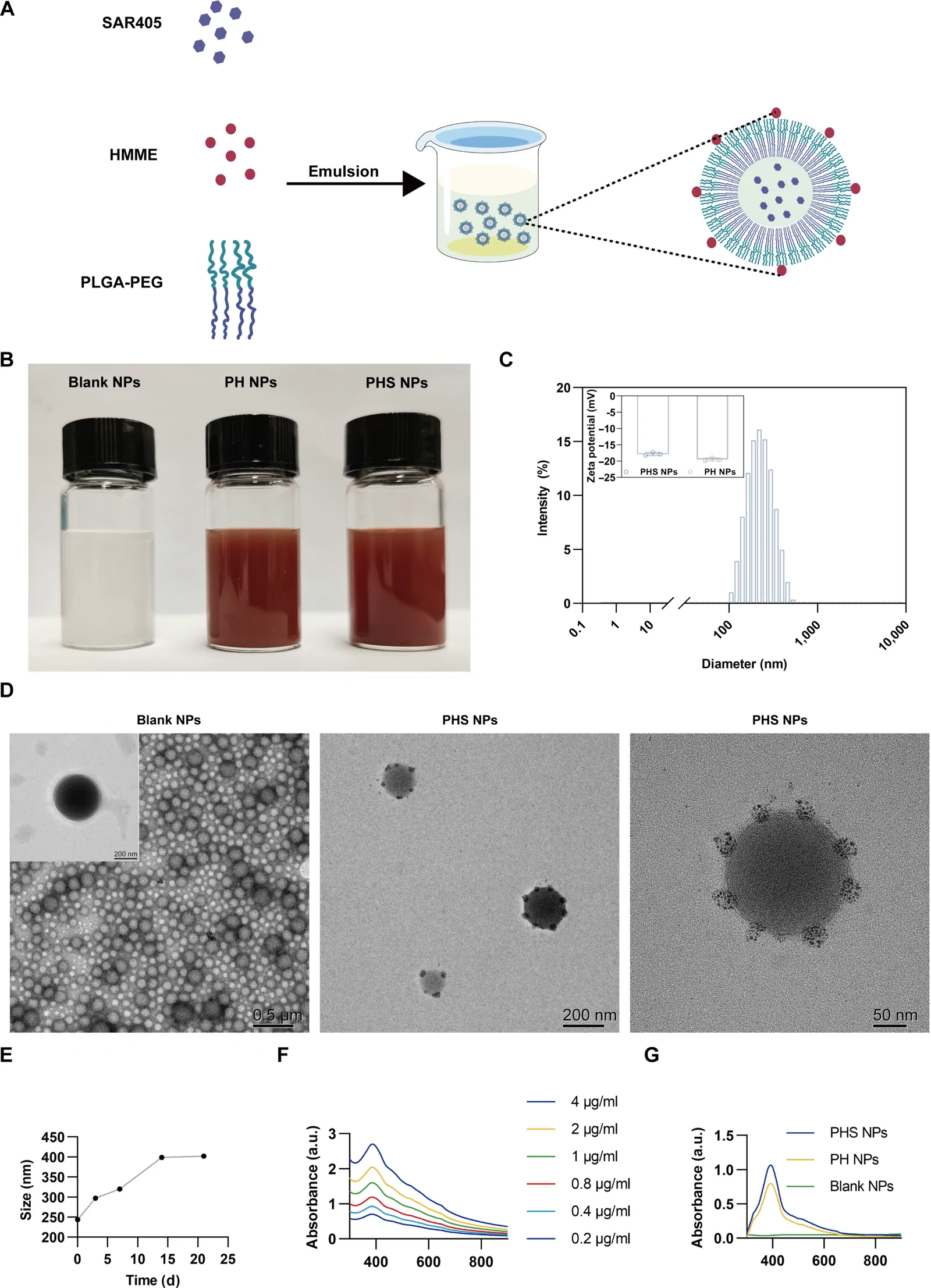
Characterization of hematoporphyrin monomethyl ether (HMME)–SAR405-loaded PLGA-PEG_2,000_ nanoparticles (PHS NPs). (A) Schematic illustration of the preparation process for PHS NPs through an emulsion method. (B) Visual appearance of aqueous dispersions: Blank NPs, PH NPs (HMME-PLGA-PEG), and PHS NPs (HMME–SAR405-PLGA-PEG) (1 mg/ml in PBS at 25 °C). Photographs taken with a mobile phone. (C) Particle size distribution of the different nanoparticle formulations measured by dynamic light scattering (DLS). (D) Transmission electron microscopy (TEM) images showing the morphology of Blank NPs and PHS NPs. Scale bars, 200 and 50 nm. (E) Temporal stability of PHS NPs, illustrating the changes in particle size over 21 d, as determined by DLS. (F) Ultraviolet–visible (UV–vis) absorption spectra of PHS NPs at varying concentrations (0.2 to 4 μg/ml). (G) UV–vis absorption spectra of different nanoparticles (Blank NPs, PH NPs, and PHS NPs) in PBS.

PHS NPs were synthesized using a 2-step emulsion method by coencapsulating the sonosensitizer HMME and the autophagy inhibitor SAR405 into PLGA-PEG_2,000_ copolymers (Fig. 2A). The PHS NPs, PH NPs, and Blank NPs showed uniform dispersion and good colloidal stability in PBS (Fig. 2B). Encapsulation of HMME gave the nanoparticle suspension a distinct reddish-brown color, differentiating it from the milky white appearance of the blank formulations. DLS measurements revealed that PHS NPs had an average hydrodynamic diameter of 213.30 ± 3.90 nm, a polydispersity index (PDI) of 0.08 ± 0.02, and a zeta potential of −18.63 ± 0.21 mV (Fig. 2C), while PH NPs exhibited a comparable hydrodynamic diameter of 226.03 ± 0.68 nm with a polydispersity index of 0.03 ± 0.03 and a zeta potential of −19.50 ± 0.45 mV, indicating good monodispersity and surface stability. TEM images (Fig. 2D) confirmed their spherical shape and uniform size distribution. Notably, dark electron-dense spots along the nanoparticle periphery suggest localized accumulation of encapsulated HMME. The long-term stability of PHS NPs was tested by measuring their size over 21 d in PBS at room temperature. The particle size increased slightly but remained below 400 nm at day 21, indicating good colloidal stability suitable for storage and systemic delivery (Fig. 2E).

To assess HMME loading and encapsulation, we used the UV–vis spectrophotometry. To determine HMME EE, we created a standard calibration curve by measuring absorbance at various concentrations (0.2 to 4 μg/ml) at 395 nm, which corresponds to HMME’s characteristic Soret band (Fig. 2F). The linear relationship between absorbance and concentration allowed reliable calculation of loading capacity (LC) and EE. The EE and LC of HMME in PHS NPs were determined to be 91.50 ± 0.36% and 8.25 ± 0.03%, respectively, based on UV–vis spectrophotometry at 395 nm. Similarly, SAR405 encapsulation was quantified using a UV–vis calibration curve over 2 to 20 μg/ml (Fig. S1), yielding an EE of 89.82 ± 1.58% and an LC of 1.45 ± 0.03%. For PH NPs, which primarily encapsulate HMME, the EE and LC were determined to be 89.50 ± 1.80% and 8.19 ± 0.13%. UV–vis spectrophotometry was performed to assess the optical properties of Blank NPs, PH NPs, and PHS NPs in PBS (Fig. 2G). Blank NPs showed no significant absorbance, while both PH NPs and PHS NPs displayed a clear peak at approximately 395 nm, corresponding to the Soret band of HMME. Although PHS NPs exhibited a higher absorbance intensity than PH NPs at 395 nm, this difference reflects variation in overall absorbance magnitude rather than the emergence of an additional spectral feature. Given the negligible intrinsic absorbance of SAR405 in this region, the observed intensity difference may be attributed to changes in the nanoparticle microenvironment following coencapsulation, including altered HMME dispersion within the polymer matrix and minor differences in light-scattering behavior. Importantly, no new absorption band was detected, and the characteristic Soret band of HMME remained clearly identifiable, supporting the reliability of HMME quantification at 395 nm. Moreover, the quantification of HMME was performed on the basis of calibration curves generated from extracted free HMME, thereby minimizing potential interference from nanoparticle-associated light scattering effects. This verifies the successful encapsulation of HMME and its maintained optical integrity during nanoparticle formulation.

### Cellular uptake, cytotoxicity, and antitumor activity of PHS NPs in vitro

**Fig. 3.**
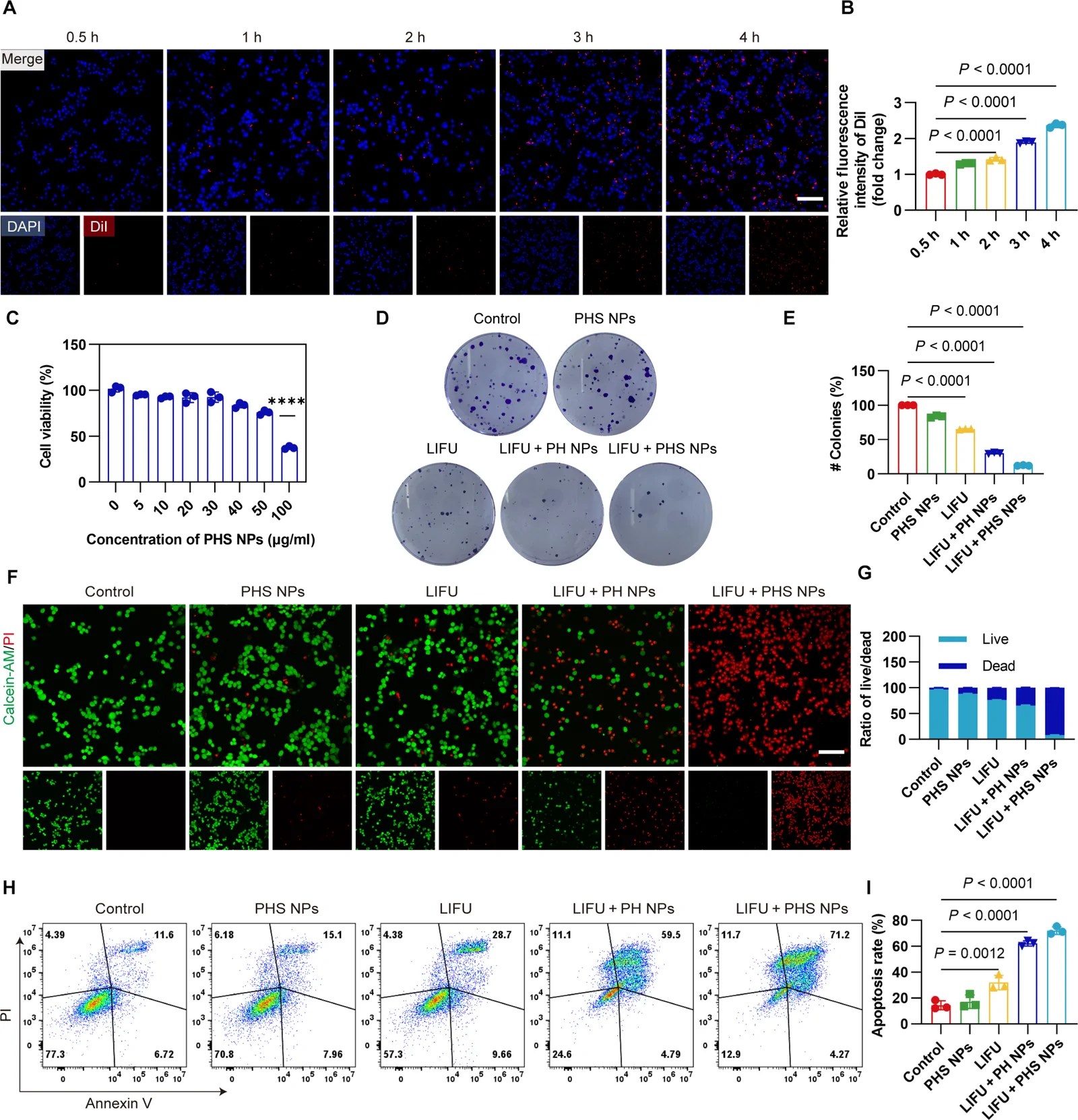
Antitumor efficacy of poly(lactic-*co*-glycolic acid)-b-poly(ethylene glycol) (PLGA-PEG_2,000_)-based nanoparticles coloaded with hematoporphyrin monomethyl ether (HMME) and SAR405 (PHS NPs) under low-intensity focused ultrasound (LIFU) irradiation in vitro. (A) Representative confocal laser scanning microscopy (CLSM) images showing cellular uptake of PHS NPs (labeled with 1,1′-dioctadecyl-3,3,3′,3′-tetramethylindocarbocyanine perchlorate [DiI]) over time (0.5 to 4 h) and quantification of fluorescence intensity (B). 4′,6-Diamidino-2-phenylindole (DAPI) (blue) stains nuclei; DiI (red) labels PHS NPs. Scale bar, 50 μm. (C) Cell viability of TC-1 cells after treatment with increasing concentrations of PHS NPs (0 to 100 μg/ml) for 24 h, measured by Cell Counting Kit-8 (CCK-8) assay. (D) Representative colony formation images under different treatments and quantitative analysis of relative colony numbers (E). (F) Representative live/dead cell staining using calcein-AM (green, viable cells) and propidium iodide (PI) (red, dead cells) visualized by CLSM and quantification of the percentage of live and dead cells (G). Scale bar, 100 μm. (H) Representative flow cytometry (FCM) analysis of apoptosis in TC-1 cells detected by annexin V–fluorescein isothiocyanate (FITC)/PI staining. (I) Quantification of the apoptosis rate (%). Data are expressed as means ± SD (*n* = 3). Statistical significance is indicated in the figure.

The intracellular uptake of PHS NPs was evaluated using CLSM and FCM. As expected, a distinct red fluorescence signal of PHS NPs (labeled with DiI) gradually appeared around the TC-1 cells after 0.5, 1, 2, 3, and 4 h of coincubation with PHS NPs (Fig. 3A and B [*P* < 0.0001]). In addition, the quantitative analysis by FCM indicates that cellular uptake is time dependent (Fig. S2), consistent with CLSM findings. To assess the biocompatibility of individual components, we examined the cytotoxicity of free HMME, SAR405, and PH NPs using the CCK-8 assay. Both agents exhibited negligible toxicity at the tested concentrations in TC-1 cells (Fig. S3), supporting their suitability for coencapsulation. In contrast, PHS NPs showed dose-dependent cytotoxicity, with a median inhibitory concentration value of 80.30 ± 2.70 μg/ml (Fig. 3C), establishing a therapeutic window for combination therapy.

The in vitro therapeutic efficacy of PHS NPs was comprehensively evaluated through colony formation assays, calcein-AM/PI live/dead staining, and annexin V–FITC/PI FCM analysis. Control and monotherapy groups (PHS NPs, 83.53 ± 1.57%; or LIFU, 65.22 ± 0.60%) retained moderate clonogenic capacity, while the LIFU + PH NP combination reduced colony formation to 30.07 ± 1.04% of the control. Notably, the LIFU + PHS NP treatment nearly abolished colony formation, leaving only 12.30 ± 0.53% relative to the control, corresponding to an 87.70% reduction (Fig. 3D and E). CLSM further confirmed this enhanced cytotoxicity following dual staining with calcein-AM and PI. Cells treated with LIFU + PHS NPs displayed prominent red fluorescence from PI, indicating extensive cell death, in contrast to the predominantly green fluorescence seen in the control and monotherapy groups, which reflects intact cell membranes and viability (Fig. 3F and G). To further quantify the mode of cell death, annexin V–FITC/PI staining was performed by FCM (Fig. 3H). A significantly higher proportion of apoptotic and necrotic cells was observed in the LIFU + PHS NPs group, confirming that the combined treatment markedly increased cell death beyond either monotherapy or the LIFU + PH NPs combination (*P* < 0.0001; Fig. 3I). This effect is likely due to ultrasound-triggered nanoparticle internalization, sonosensitizer-mediated ROS production, and SAR405-induced autophagy inhibition, working together to induce tumor cell apoptosis.

In summary, these findings highlight the potent and synergistic antitumor effects of PHS NPs combined with LIFU in vitro, presenting a rational therapeutic strategy that integrates targeted drug delivery, SDT, and modulation of autophagy.

### Mitochondrial damage and autophagic flux disruption

**Fig. 4.**
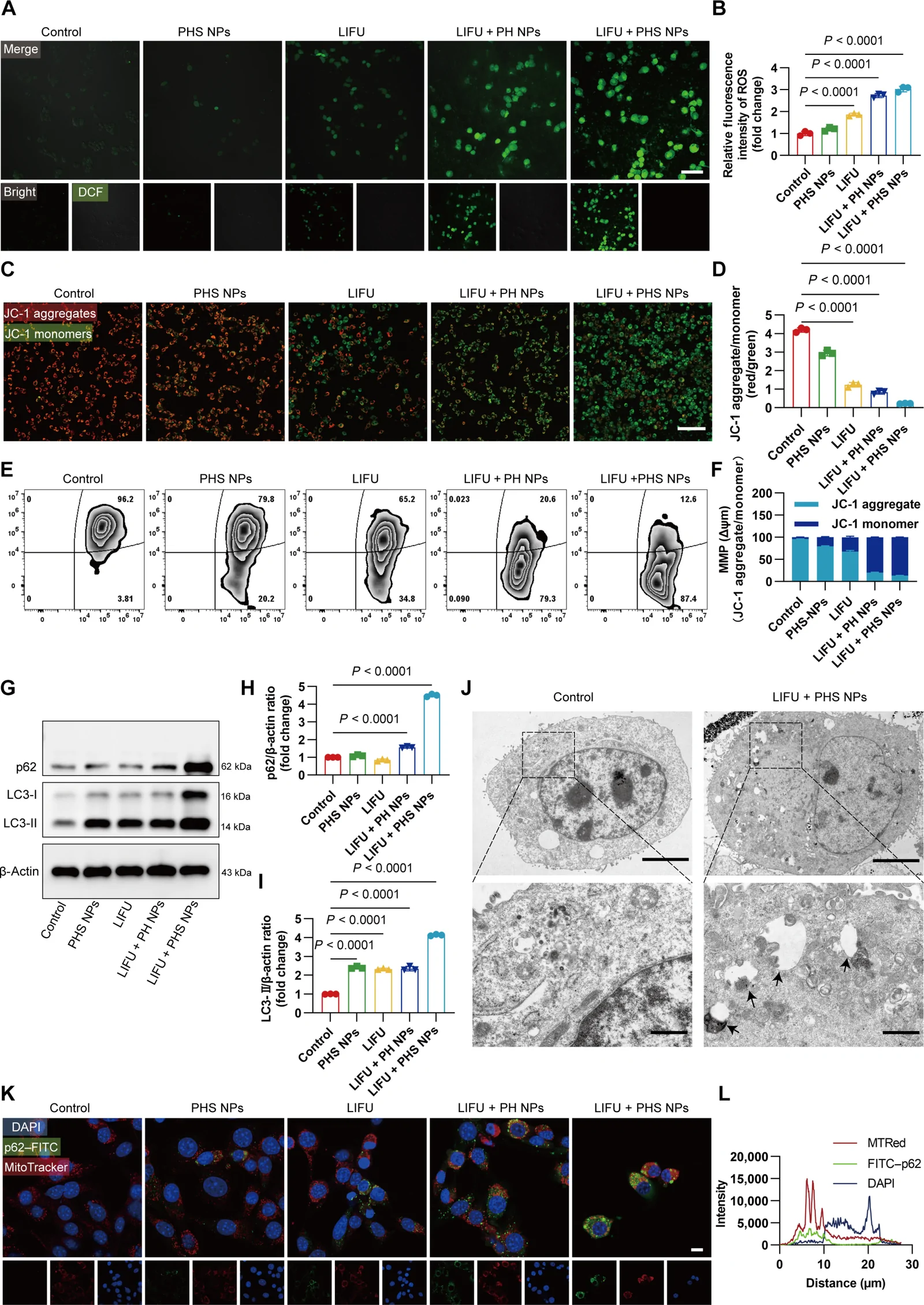
Evaluation of mitochondrial function and inhibition of autophagy in TC-1 cells treated with poly(lactic-*co*-glycolic acid)-b-poly(ethylene glycol) (PLGA-PEG_2,000_)-based nanoparticles coloaded with hematoporphyrin monomethyl ether (HMME) and SAR405 (PHS NPs) under low-intensity focused ultrasound (LIFU) irradiation. (A) Representative confocal laser scanning microscopy (CLSM) images of intracellular reactive oxygen species (ROS) generation visualized by 2′,7′-dichlorodihydrofluorescein diacetate (DCFH-DA) (green) merged with bright-field images, with corresponding quantification of mean fluorescence intensity (B). Scale bar, 100 μm. (C) Representative JC-1 staining of ΔΨm in TC-1 cells and quantification of the red/green fluorescence intensity ratio (D). Scale bar, 50 μm. (E) Representative flow cytometry (FCM) of ΔΨm using JC-1 and quantitative analysis (F). (G) Western blot analysis of autophagy-related proteins and quantification of microtubule-associated protein 1A/1B–light chain 3-II (LC3-II)/β-actin (H) and p62/β-actin (I) ratios. (J) Transmission electron microscopy (TEM) images showing autophagosome- and autolysosome-like vesicles in TC-1 cells. Control cells display normal ultrastructure, whereas LIFU + PHS NPs–treated cells exhibit numerous vesicles (black arrows). Scale bars, 5 and 1 μm. (K) Representative CLSM images showing colocalization of p62 (green) and MitoTracker (red) (MTRed) in TC-1 cells. 4′,6-diamidino-2-phenylindole (DAPI) (blue) stains nuclei. Scale bar, 10 μm. (L) Line-scan analysis of fluorescence intensity. Data are expressed as means ± SD (*n* = 3). Statistical significance is indicated in the figure.

The intracellular ROS level was measured using the fluorescent probe DCFH-DA to assess the sonodynamic efficacy of various treatments in TC-1 cancer cells. Bright-green fluorescence appeared in LIFU and LIFU + PHS NP-treated TC-1 cells, with stronger fluorescence in the latter (*P* < 0.0001; Fig. 4A and B). Notably, the green fluorescence intensity in the PHS NPs group was higher than in the LIFU group. It was diffusely distributed throughout the intracellular region, indicating significant ROS generation even without LIFU irradiation. This increase in ROS observed in the PHS NPs group may be associated with SAR405-mediated modulation of autophagic degradation, which could limit the clearance of oxidatively damaged components and contribute to intracellular ROS accumulation. FCM further confirmed that combining PHS NPs with LIFU irradiation produced the most substantial increase in intracellular ROS, markedly surpassing that seen in the other groups (*P* < 0.0001; Fig. S4). To further characterize ROS species, we used DHE to detect superoxide anion (O_2_·^−^). FCM showed that DHE fluorescence was markedly elevated in the LIFU + PHS NPs group compared to the control, LIFU-only, and PHS NP-only groups (*P* < 0.0001; Fig. S5), confirming the generation of superoxide anion upon ultrasound activation.

Given the close association between ROS accumulation and mitochondrial damage, ΔΨm was next evaluated using the JC-1 probe. In the control and PHS NPs groups, cells exhibited predominantly red fluorescence, indicative of intact ΔΨm. Upon LIFU treatment, a partial shift from red to green fluorescence was observed, suggesting moderate mitochondrial depolarization. Notably, the LIFU + PHS NPs group displayed the most pronounced green fluorescence with markedly reduced red signal (Fig. 4C), indicating severe loss of ΔΨm. Quantitative analysis of the red/green fluorescence intensity ratio revealed a significant decrease in ΔΨm in the LIFU + PHS NPs group compared with all other treatments (*P* < 0.0001; Fig. 4D). These observations were corroborated by FCM, which showed that the LIFU + PHS NPs group had the highest proportion of cells with disrupted ΔΨm compared to all other groups (Fig. 4E and F). To further assess mitochondrial functional impairment, we quantified the intracellular adenosine triphosphate (ATP) levels. Consistent with the observed mitochondrial depolarization, LIFU treatment caused a moderate reduction in intracellular ATP levels, whereas the LIFU + PHS NPs group exhibited the most pronounced ATP depletion (*P* < 0.0001; Fig. S6), indicating severe disruption of mitochondrial bioenergetics and energy metabolism.

To clarify how PHS NPs and LIFU affect autophagic flux, we used Western blotting, TEM, and CLSM. In the LIFU group, Western blot results showed increased LC3-II levels without a corresponding rise in p62, indicating that autophagy was activated and autophagic flux remained functional. Conversely, the LIFU + PHS NPs group exhibited a notable accumulation of both LC3-II and p62 (Fig. 4G [*P* < 0.0001], H, and I), indicating that although autophagosome formation was enhanced, the subsequent degradation process was inhibited. This was supported by a SAR405 dose–response experiment, where higher concentrations of this phosphatidylinositol 3-kinase class III inhibitor caused a dose-dependent increase in p62 protein, confirming p62 as a marker for impaired autophagic degradation (Fig. S7).

Further evidence was obtained from TEM, which revealed an accumulation of enlarged autolysosomes containing undigested cellular contents in the LIFU + PHS NPs group compared to the controls (Fig. 4J), indicating defective autophagic cargo clearance. Similarly, CLSM analysis of p62–FITC and MitoTracker Red CMXRos staining showed markedly increased fluorescence intensity of p62 in proximity to damaged mitochondria in the LIFU + PHS NPs group (Fig. 4K [*P* < 0.0001] and Fig. S8). This colocalization was further supported by line-scan analysis of fluorescence intensity (Fig. 4L), suggesting mitochondria-associated autophagic targeting under SDT stress conditions. These findings indicate that damaged mitochondria were labeled for autophagic processing but were not efficiently degraded, consistent with impaired lysosome-dependent autophagic clearance. Furthermore, immunofluorescence of LC3B showed increased punctate autophagosome accumulation in the LIFU + PHS NPs group compared to controls and PHS NPs alone (Fig. S9), indicating enhanced autophagosome formation accompanied by flux blockade. The weaker LC3B signal in the PHS NPs group likely results from limited sonosensitizer release without ultrasound activation.

In addition, LAMP1 immunofluorescence revealed decreased lysosomal membrane protein expression (Fig. S10), and LysoSensor staining demonstrated reduced lysosomal acidification (Fig. S11), both indicative of lysosomal dysfunction. The simultaneous accumulation of LC3-II and p62, together with ultrastructural evidence of undigested autolysosomes, supports lysosomal degradation failure as a principal contributor to autophagic flux disruption in the LIFU + PHS NPs group. This impairment is likely associated with excessive ROS generated by LIFU-activated PHS NPs, which may compromise lysosomal integrity and enzymatic activity. Under these conditions, autophagic cargo degradation is hindered, resulting in the accumulation of autophagosomes and damaged mitochondria. Taken together, these molecular, structural, and imaging findings support the conclusion that LIFU + PHS NPs are associated with activation of mitochondrial-related autophagic responses while concurrently impairing lysosome-dependent degradation, thereby promoting sustained intracellular stress.

Overall, these results indicate that lysosome-associated autophagic flux represents a key regulatory node influencing SDT-induced mitochondrial stress and cytotoxic response. While further studies may help to delineate the broader regulatory network involved, our findings highlight stage-specific disruption of lysosomal autophagic flux as a critical determinant of therapeutic outcome.

### Immune activation in vitro

ICD can induce strong antitumor immune responses by releasing danger-associated molecular patterns, such as surface-exposed CRT, HMGB1, and extracellular ATP, which collectively contribute to the modulation of the tumor microenvironment [49,50].

**Fig. 5.**
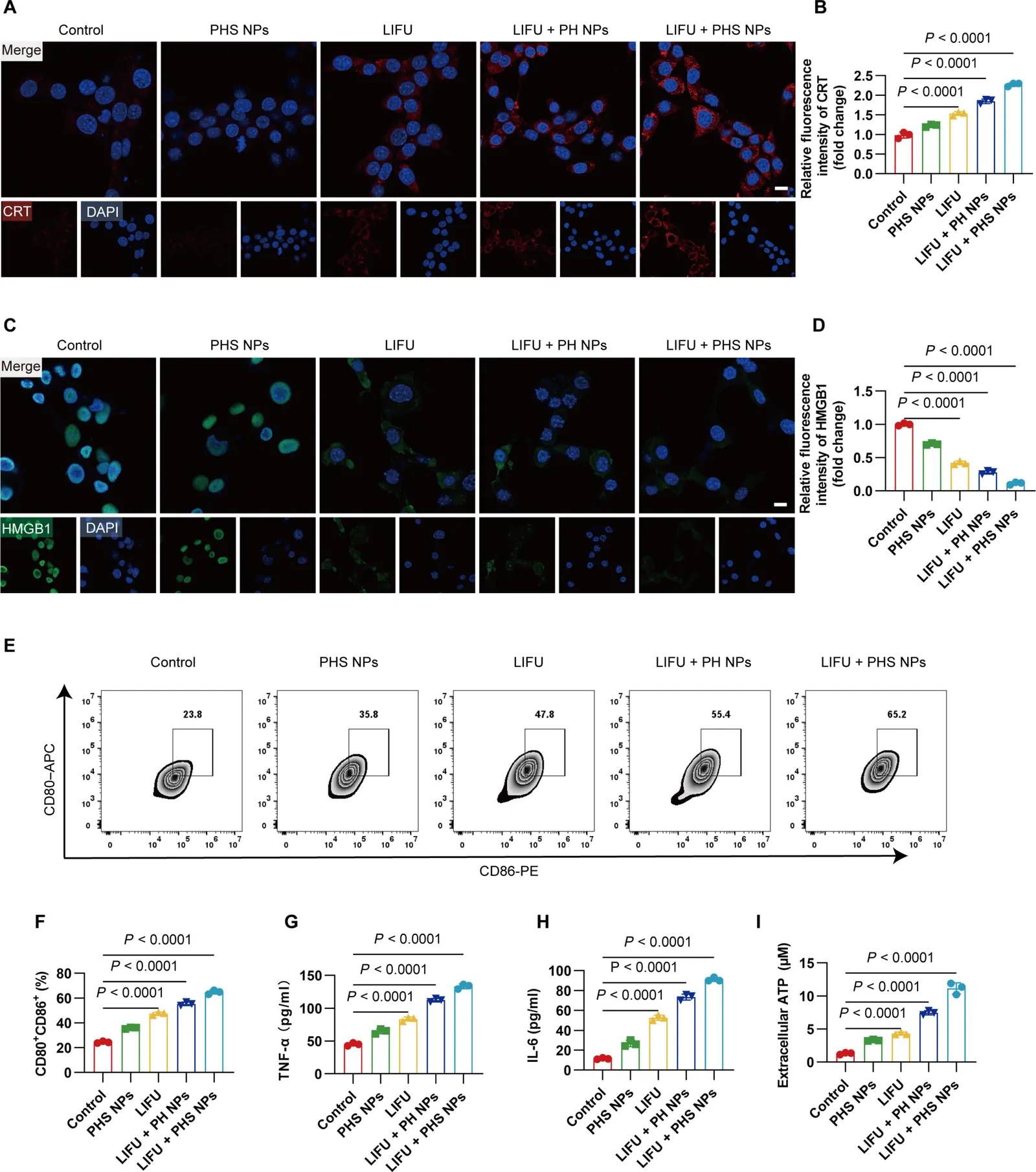
Evaluation of immunogenic cell death (ICD) and immune response in vitro. (A) Representative confocal laser scanning microscopy (CLSM) images showing calreticulin (CRT) exposure on the cell surface as a marker of ICD and quantification of fluorescence intensity (B). CRT–Cy3 (red) and 4′,6-diamidino-2-phenylindole (DAPI) (blue). Scale bar, 10 μm. (C) Representative CLSM images showing high-mobility group box 1 (HMGB1) release and quantification of fluorescence intensity (D). HMGB1–fluorescein isothiocyanate (FITC) (green) and DAPI (blue). Scale bar, 10 μm. (E) Representative flow cytometry (FCM) of dendritic cell (DC) maturation (CD80^+^CD86^+^ surface expression) and quantification (F). (G and H) Enzyme-linked immunosorbent assay (ELISA) quantification of tumor necrosis factor-α (TNF-α) and interleukin-6 (IL-6) levels in the supernatant. (I) Extracellular adenosine triphosphate (ATP) levels in culture supernatants following different treatments. Data are expressed as means ± SD (*n* = 3). Statistical significance is indicated in the figure.

To evaluate the ICD-inducing capacity of PHS NPs under LIFU irradiation, we first examined CRT translocation to the cell surface and HMGB1 release. CLSM images displayed bright-red fluorescence, indicating CRT exposure on the plasma membrane in the LIFU + PHS NPs group, demonstrating effective ICD induction by SDT (Fig. 5A). At the same time, the reduction in green nuclear fluorescence of HMGB1 suggested its movement to the cytoplasm and subsequent release outside the cell (Fig. 5C). Notably, the LIFU-only and PHS NP-only groups showed limited CRT expression and minimal HMGB1 release (*P* < 0.0001; Fig. 5B and D), indicating that LIFU alone can trigger minor stress responses but is insufficient for robust ICD. Importantly, extracellular ATP levels in culture supernatants were significantly elevated in the LIFU + PHS NPs group compared with the control and LIFU-only groups, supporting ATP release into the extracellular space as a hallmark of ICD (*P* < 0.0001; Fig. 5I).

To further assess whether these ICD markers could stimulate innate immune activation, we used a Transwell coculture system to observe the responses of DCs and macrophages. TC-1 cells treated under different conditions were cocultured with DC2.4 and RAW 264.7 cells. After 12 h, FCM showed that LIFU + PHS NPs treatment resulted in a significantly higher proportion of CD80^+^CD86^+^ mature DCs and CD86^+^CD206^−^ M1-polarized macrophages compared to the untreated, LIFU-only, or PHS NPs-only groups (*P* < 0.0001; Fig. 5E and F and Fig. S12). In addition, ELISA revealed a notable increase in TNF-α and IL-6 secretion in the supernatants of the LIFU + PHS NPs group (*P* < 0.0001; Fig. 5G and H), indicating a strong proinflammatory and immune-activating response.

These findings indicate that while LIFU alone induces minor ICD-related signals, the combination of LIFU + PHS NPs produces a marked ICD response associated with increased DC maturation, M1 macrophage polarization, and proinflammatory immune activation in vitro.

### Biosafety of the PHS NPs in vivo

**Fig. 6.**
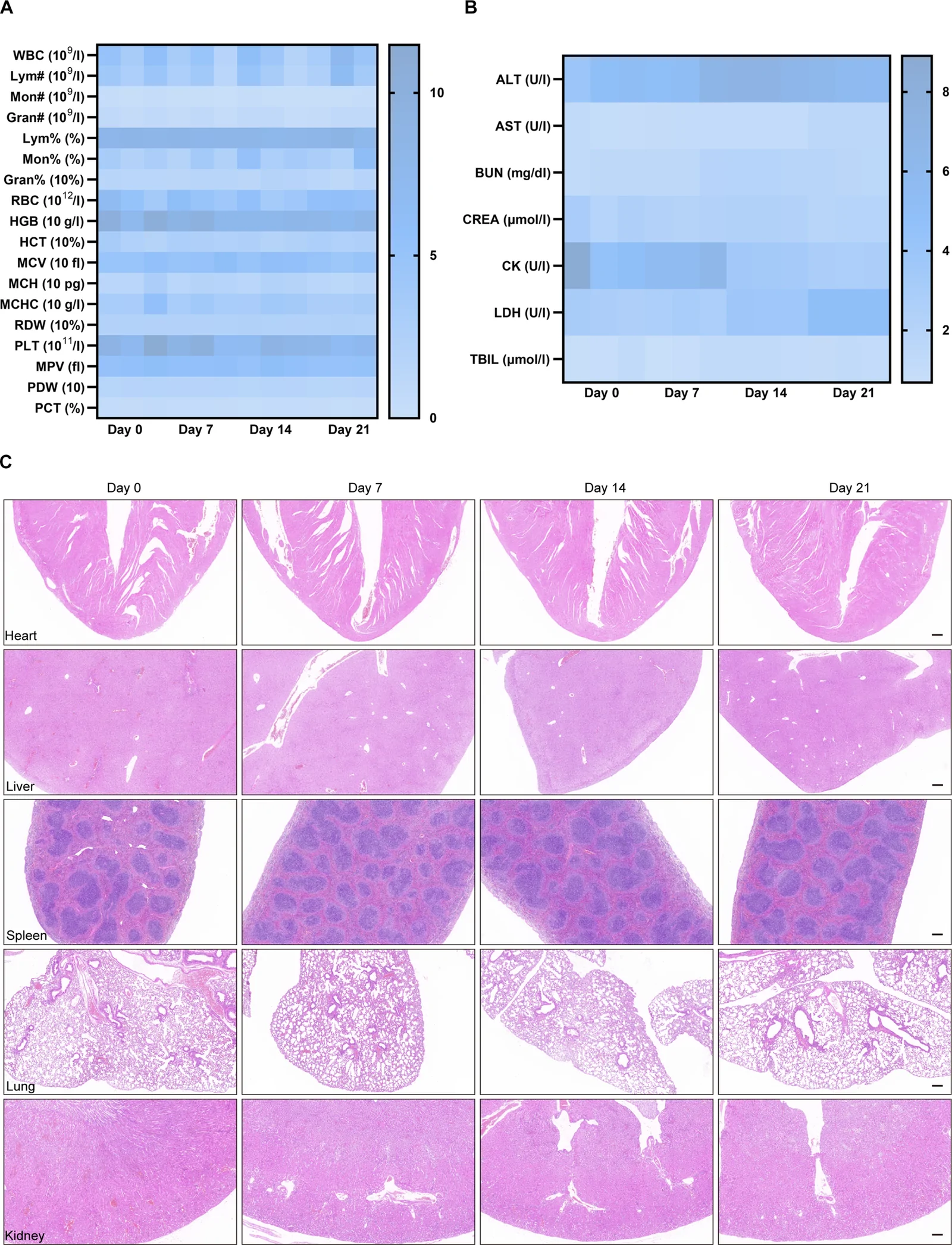
Biosafety of poly(lactic-co-glycolic acid)-b-poly(ethylene glycol) (PLGA-PEG_2,000_)-based nanoparticles coloaded with hematoporphyrin monomethyl ether (HMME) and SAR405 (PHS NPs) in vivo. (A) Heatmap depicting the changes in hematological parameters, including white blood cell (WBC), lymphocyte count (Lym), monocyte count (Mon), granulocyte count (Gran), red blood cell (RBC) count, hemoglobin (HGB), hematocrit (HCT), mean corpuscular volume (MCV), mean corpuscular hemoglobin (MCH), mean corpuscular hemoglobin concentration (MCHC), red cell distribution width (RDW), platelet count (PLT), mean platelet volume (MPV), platelet distribution width (PDW), plateletcrit (PCT), and other related markers, measured at days 0, 7, 14, and 21. (B) Heatmap illustrating the serum biochemical changes over time, including alanine aminotransferase (ALT), aspartate aminotransferase (AST), blood urea nitrogen (BUN), creatinine (CREA), creatine kinase (CK), lactate dehydrogenase (LDH), and total bilirubin (TBIL) levels at days 0, 7, 14, and 21. (C) Hematoxylin and eosin (H&E) staining of major organs (heart, liver, spleen, lung, and kidney) at days 0, 7, 14, and 21. Scale bars, 200 μm.

An ideal nanocarrier should have well-defined physicochemical properties, minimal toxicity, and high biocompatibility. To evaluate the in vivo biosafety of PHS NPs, we performed comprehensive hematological and histopathological assessments in C57BL/6 mice following intravenous injection. Hematological parameters and serum biochemical indices were monitored over time at days 0, 7, 14, and 21 after injection. Complete blood count analysis revealed no significant fluctuations in red blood cell (RBC) or white blood cell (WBC) counts among the treated groups, indicating no hematological toxicity (Fig. 6A). Serum biochemical analysis also demonstrated stable levels of hepatic (ALT and AST), renal (BUN and creatinine), and myocardial (LDH and CK) function markers across all time points, remaining within normal physiological ranges (Fig. 6B). These data suggest the absence of systemic toxicity following PHS NP administration.

Furthermore, histological analysis of major organs (heart, liver, spleen, lung, and kidney) by H&E staining showed preserved tissue architecture with no signs of inflammation, necrosis, or pathological alterations, both in the short and long term (Fig. 6C).

Overall, these results show that PHS NPs have excellent in vivo biocompatibility and minimal systemic toxicity, supporting their potential as a safe and clinically applicable SDT platform.

### In vivo antitumor efficacy of autophagy inhibition/SDT synergy

**Fig. 7.**
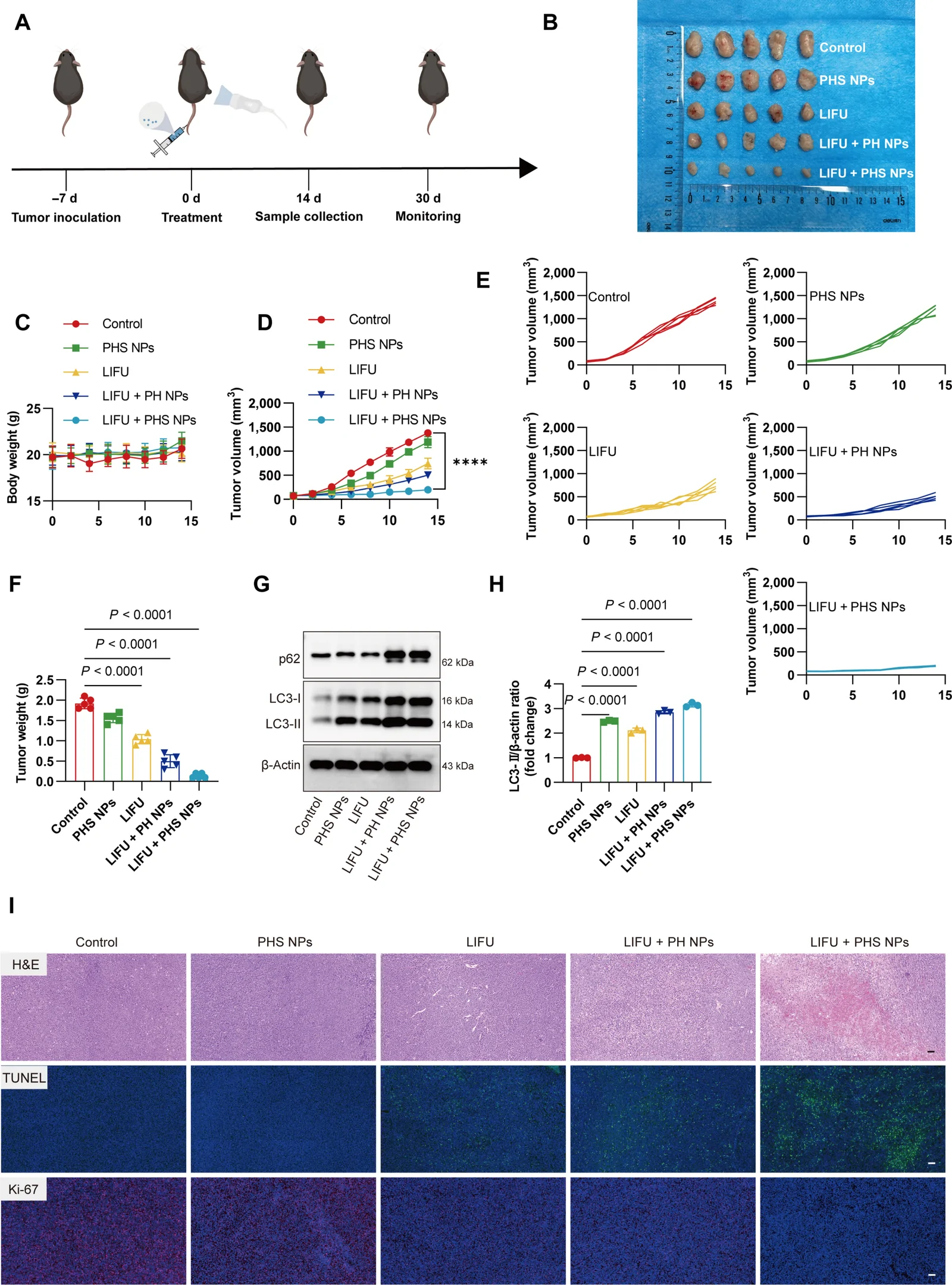
In vivo antitumor synergy of sonodynamic therapy (SDT) and autophagy inhibition under different treatments. (A) Schematic illustration of the experimental workflow, including the TC-1 tumor model and the therapeutic regimen for evaluating in vivo antitumor efficacy. (B) Representative photographs of harvested tumors at day 14 posttreatment. The low-intensity focused ultrasound (LIFU) + poly(lactic-co-glycolic acid)-b-poly(ethylene glycol) (PLGA-PEG_2,000_)-based nanoparticles coloaded with hematoporphyrin monomethyl ether (HMME) and SAR405 (PHS NPs) group showed markedly smaller tumor size compared to other groups. (C) Body weight changes show no significant weight loss, indicating low systemic toxicity. (D) Tumor volume growth curves. Mice treated with LIFU + PHS NPs exhibited near-complete tumor growth suppression. Statistical significance was determined based on endpoint tumor volume on day 14 only (****P < 0.0001). (E) Individual tumor growth trajectories for each mouse. Mice in the LIFU + PHS NPs group showed minimal tumor progression. (F) Tumor weight analysis at day 14. Significant tumor mass reduction was observed in the LIFU + PHS NPs group. (G) Western blot analysis of autophagy-related proteins and quantitative analysis of microtubule-associated protein 1A/1B–light chain 3-II (LC3-II)/β-actin ratios (H). (I) Histological and immunofluorescent staining of tumor sections. Hematoxylin and eosin (H&E) staining showed reduced cellularity in the combination group. Terminal deoxynucleotidyl transferase-mediated deoxyuridine triphosphate nick end labeling (TUNEL) staining revealed enhanced apoptosis (green), while Ki-67 staining confirmed suppressed proliferation (red). Scale bars, 50 μm (H&E), 100 μm (TUNEL), and 100 μm (Ki-67). Tumor growth, body weight, and tumor weight data are shown for *n* = 5 mice per group. Western blot analyses were performed using *n* = 3 mice per group, and representative histological/immunofluorescence images are shown. Data are expressed as means ± SD (*n* = 3). Statistical significance is indicated in the figure.

Motivated by the improved autophagy blockade and antitumor effects observed in vitro, we further assessed the ability of PHS NPs to inhibit tumor growth in vivo. As shown in the experimental procedure (Fig. 7A), tumor-bearing C57BL/6 mice were randomly divided into 5 groups to receive different treatments, with the mice’s weight and tumor volume monitored every other day. The control and LIFU groups exhibited substantial tumor burdens, whereas a moderate reduction was observed in the PHS NPs group. Notably, the LIFU + PHS NPs group demonstrated the most significant tumor regression, emphasizing the synergistic antitumor efficacy of the combined treatment (Fig. 7B).

Similarly, body weight monitoring (Fig. 7C) showed no significant differences among groups throughout the treatment period, indicating favorable biocompatibility and minimal systemic toxicity of the interventions. Tumor volume curves (Fig. 7D) further substantiate these observations. Tumors in the LIFU and PHS NPs group showed rapid progression, with an 11.7-fold and 16.1-fold increase over baseline by day 14, confirming that LIFU and PHS NPs alone had minimal therapeutic benefit. In contrast, the LIFU + PH NPs group exhibited moderate tumor growth inhibition (around 6.18-fold increase), likely due to sonodynamic antitumor effects. Strikingly, the LIFU + PHS NPs group demonstrated almost complete suppression of tumor growth throughout the treatment period, underscoring the potent synergistic effect of the combined therapy. Furthermore, to evaluate individual tumor responses, we plotted the growth curves for each mouse (Fig. 7E). While mice in the control, LIFU, and PHS NPs groups exhibited varying levels of tumor progression, mice treated with LIFU + PHS NPs consistently maintained low tumor volumes throughout the study, with no evidence of relapse, highlighting the reliability and long-lasting efficacy of the synergistic therapy. Quantitative analysis of excised tumor weights (Fig. 7F) was consistent with the tumor volume data, with tumors from the LIFU + PHS NPs group being significantly lighter than those from the other groups, confirming the superior therapeutic efficacy of the combination treatment.

To explore the underlying mechanisms, we performed the Western blot analysis on tumor lysates (Fig. 7G). The results showed substantial accumulation of both LC3-II and p62 in tumors from the PHS NPs and LIFU + PHS NPs groups, indicative of effective autophagic flux blockade. Specifically, LC3-II expression increased by approximately 3.15-fold in the LIFU + PHS NPs group compared to the control (*P* < 0.0001; Fig. 7H). Similarly, p62 expression was markedly up-regulated by about 2.04-fold. These findings strongly support that autophagy inhibition plays a central role in mediating the enhanced antitumor efficacy of the combination treatment (*P* < 0.0001; Fig. S13).

Histopathological and immunohistochemical analyses provided further evidence of the therapeutic efficacy of the combined treatment. H&E staining of major organs (heart, liver, spleen, lungs, and kidneys) revealed no observable structural abnormalities in the LIFU + PHS NPs group, indicating favorable biosafety (Fig. S14). In contrast, H&E staining of tumor sections demonstrated extensive structural disruption and cellular disintegration in the LIFU + PHS NPs group, consistent with severe necrosis and apoptosis induced by the synergistic effects of SDT and autophagy inhibition (Fig. 7I). Supporting this observation, TUNEL staining showed a marked increase in green fluorescence in the combination group, indicating substantially higher levels of tumor cell apoptosis than in the other groups. Notably, the LIFU-only group exhibited a minor number of TUNEL-positive cells, reflecting a limited apoptotic effect of ultrasound alone, whereas the LIFU + PHS NPs group showed markedly enhanced apoptosis, highlighting the necessity of nanoparticle-mediated SDT for effective tumor cell killing. In addition, Ki-67 immunohistochemistry demonstrated significantly reduced proliferative activity in tumors treated with LIFU + PHS NPs, with the lowest Ki-67 expression observed among all groups, further confirming the superior efficacy of the combination therapy. Together, these results corroborate that the combinatorial treatment not only suppresses tumor growth but also effectively induces apoptosis and inhibits proliferation at the histological level.

The combined treatment of LIFU and PHS NPs resulted in significant tumor growth inhibition, accompanied by a marked accumulation of LC3-II and p62 in tumor tissues, indicating effective blockade of autophagic flux. These findings suggest that the enhanced antitumor efficacy observed in vivo is closely associated with impaired autophagic degradation. Such a blockade may exacerbate intracellular stress, thereby increasing tumor cell susceptibility to SDT-induced cytotoxicity. Taken together, these results support the contribution of lysosome-dependent autophagic flux disruption to the enhanced therapeutic efficacy of the combination strategy in vivo.

### Immune activation in vivo

**Fig. 8.**
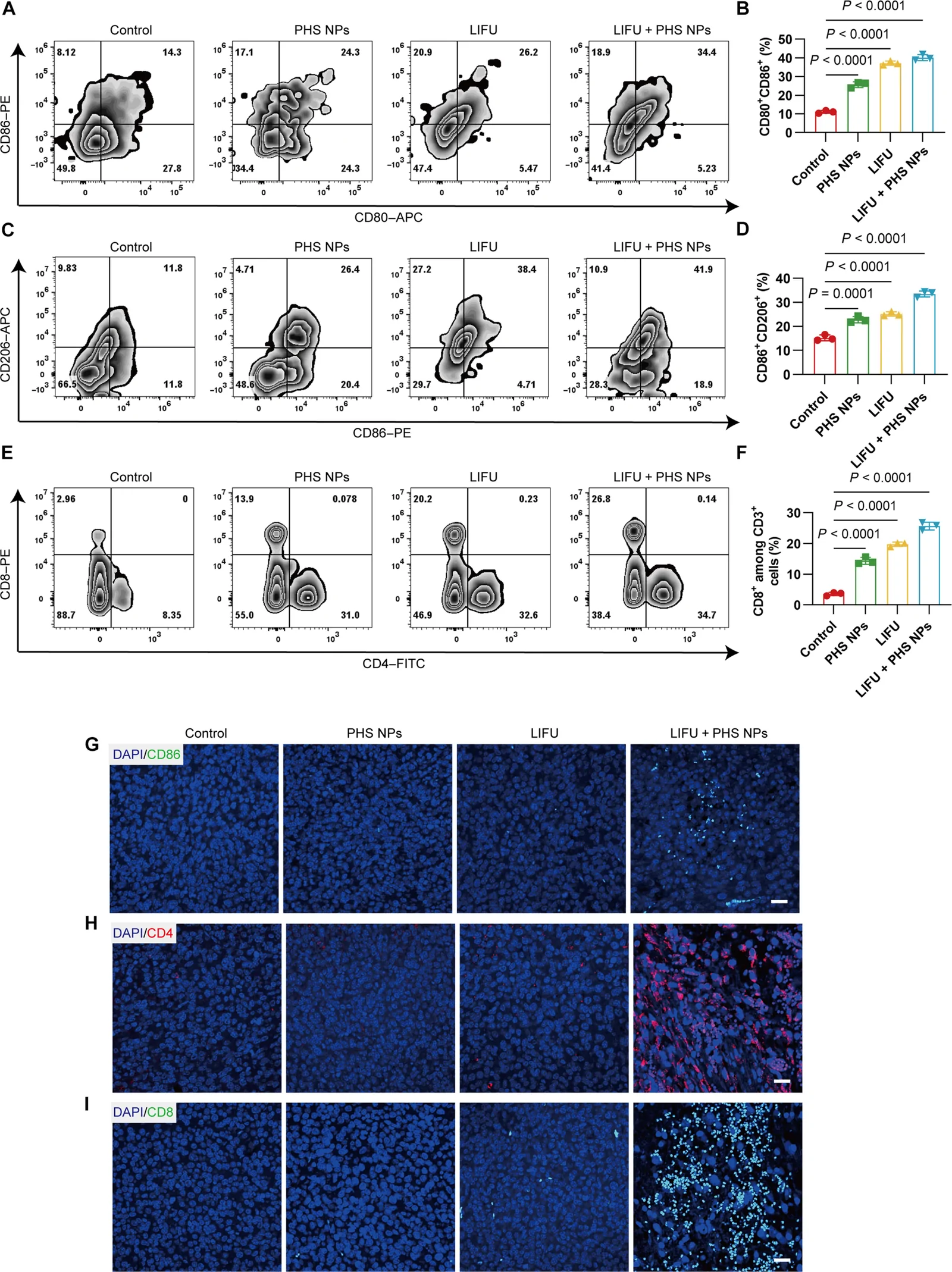
Immune activation in tumor tissues and spleens following different treatments. (A) Representative flow cytometry (FCM) plots and (B) quantitative analysis of CD80^+^/CD86^+^ mature dendritic cells (DCs) in the tumor microenvironment. (C) Representative FCM plots and (D) quantification of macrophage polarization assessed by CD86^+^ (M1) versus CD206^+^ (M2) expression in tumor tissues. (E) Representative FCM plots and (F) quantitative analysis of CD4^+^ and CD8^+^ T cell infiltration in spleens. (G to I) Immunofluorescence staining of tumor sections showing the distribution of CD86^+^ macrophages (G), CD4^+^ helper T cells (H), and CD8^+^ cytotoxic T cells (I) in control, low-intensity focused ultrasound (LIFU), poly(lactic-*co*-glycolic acid)-b-poly(ethylene glycol) (PLGA-PEG_2,000_)-based nanoparticles coloaded with hematoporphyrin monomethyl ether (HMME) and SAR405 (PHS NPs), and LIFU + PHS NPs treatment groups. Cell nuclei were stained with 4′,6-diamidino-2-phenylindole (DAPI) (blue). Scale bars, 20 μm. Data are expressed as means ± SD (*n* = 3). Statistical significance is indicated in the figure.

FCM was used to assess immune activation in tumor tissues and spleens following various treatments. In the LIFU + PHS NPs group, a significant rise in mature DCs (CD11c^+^CD80^+^CD86^+^) and M1-polarized macrophages (F4/80^+^CD86^+^) was observed (*P* < 0.0001; Fig. 8A to D), indicating enhanced antigen presentation and a shift toward a proinflammatory phenotype. These changes suggest enhanced activation of innate immune components, which are crucial for initiating downstream adaptive responses. In addition, the number of CTLs (CD3^+^CD8^+^) was markedly higher in spleens (*P* < 0.0001; Fig. 8E and F), along with an increase in helper T cells (CD4^+^), indicating successful immune priming and activation. This coordinated response, characterized by DC maturation, M1 polarization, and CTL expansion, suggests that LIFU + PHS NPs treatment is associated with modulation of the tumor microenvironment and systemic immune activation, thereby promoting enhanced antitumor immune responses. Consistent with FCM data, immunofluorescence results showed a significant up-regulation of key immune effectors in LIFU + PHS NPs-treated tumors (Fig. 8G to I). Specifically, CD86 fluorescence intensity (a marker of APC activation) was markedly increased. Similarly, CD4 and CD8 signals were elevated, indicating enhanced infiltration of helper and cytotoxic T cells within tumor tissues. These coordinated increases across innate and adaptive immune markers further support the association between combination treatment and remodeling of the tumor immune microenvironment.

Together, these complementary analyses indicate that PHS NPs combined with LIFU exert a dual antitumor effect: direct tumor cell destruction via sonodynamic damage and modulation of the tumor immune microenvironment, thereby enhancing antitumor immunity.

## Conclusion

This work presents PHS NPs as a multifunctional nanoplatform integrating SDT with selective autophagy modulation for tumor treatment. Upon LIFU irradiation, PHS NPs generate ROS, inducing mitochondrial and lysosomal stress, while concurrently releasing SAR405 to interfere with VPS34-dependent autophagic flux. The coordinated action of ROS generation and modulation of autophagic flux was associated with LC3-II and p62 accumulation and the presence of undegraded autolysosomal structures, supporting the notion that lysosome-dependent autophagic flux may contribute to cytoprotective adaptation during SDT. Both in vitro and in vivo studies showed that combining SDT with autophagic flux modulation was associated with enhanced tumor suppression and increased markers of ICD. Moreover, treatment was accompanied by modulation of the tumor immune microenvironment, including macrophage polarization toward an M1-like phenotype and increased cytotoxic immune infiltration. Importantly, beyond the general concept of autophagy modulation to enhance SDT, this study highlights stage-resolved features of lysosome-associated autophagic flux under SDT-induced stress conditions.

Overall, these findings suggest that synchronized nanocarrier-mediated codelivery of a sonosensitizer and a VPS34 inhibitor may represent a rational strategy to mitigate SDT-associated adaptive resistance and improve antitumor immune responsiveness in HPV-associated tumors.

## Data Availability

The data supporting this study’s findings are available from the corresponding author upon reasonable request.
