# Supplementary material for: Autophagy-Modulated Sonodynamic Therapy Triggers Mitochondrial Catastrophe for Potent Immunogenic Tumor Eradication
Source: Biomater Res. 2026 Apr 15;30:0348. doi: 10.34133/bmr.0348 (PMC13081276; doi:10.34133/bmr.0348)
Supplement: Supplementary 1 — Figs. S1 to S14 [file bmr.0348.f1.doc]

# **SUPPLEMENTARY MATERIALS**

Title

Autophagy-Modulated Sonodynamic Therapy Triggers Mitochondrial Catastrophe for Potent Immunogenic Tumor Eradication

**Authors**

Mengmeng Li^1^†, Hua Song^2^†, Ya Zhu^3^†, Wei Zhang^4*^, Tianan Jiang^1*^

**Affiliations**

1. Department of Ultrasound Medicine, The First Affiliated Hospital, College of Medicine, Zhejiang University, Hangzhou,310003 China.
2. Department of General Surgery, Daping Hospital, Third Military Medical University, Chongqing, 400042 China.
3. Department of Obstetrics and Gynecology, Chongqing Traditional Chinese Medicine Hospital, Jiulongpo District, Chongqing, 400000 China.
4. Department of Ultrasound Medicine, the Second Affiliated Hospital of Chongqing Medical University & Chongqing Key Laboratory of Ultrasound Molecular Imaging, Chongqing,400012 China.

*Address correspondence to: Wei Zhang; 306902@hospital.cqmu.edu.cn and Tianan Jiang; [tiananjiang@zju.edu.cn](mailto:tiananjiang@zju.edu.cn)

†These authors contributed equally to this work

**Supplementary Figures**


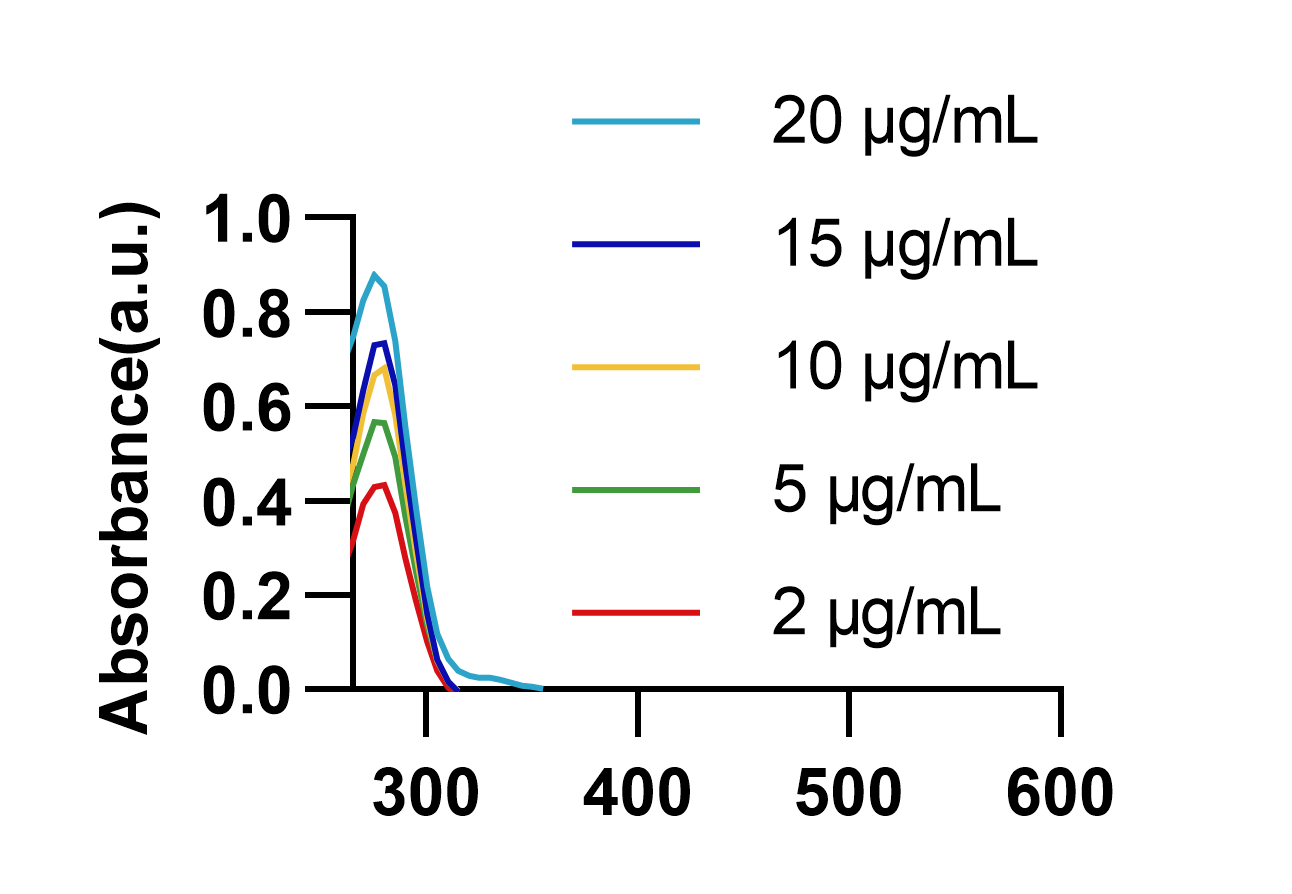


Fig. S1. UV-vis absorption spectra of PHS NPs at varying concentrations (2 µg/mL to 20 µg/mL).


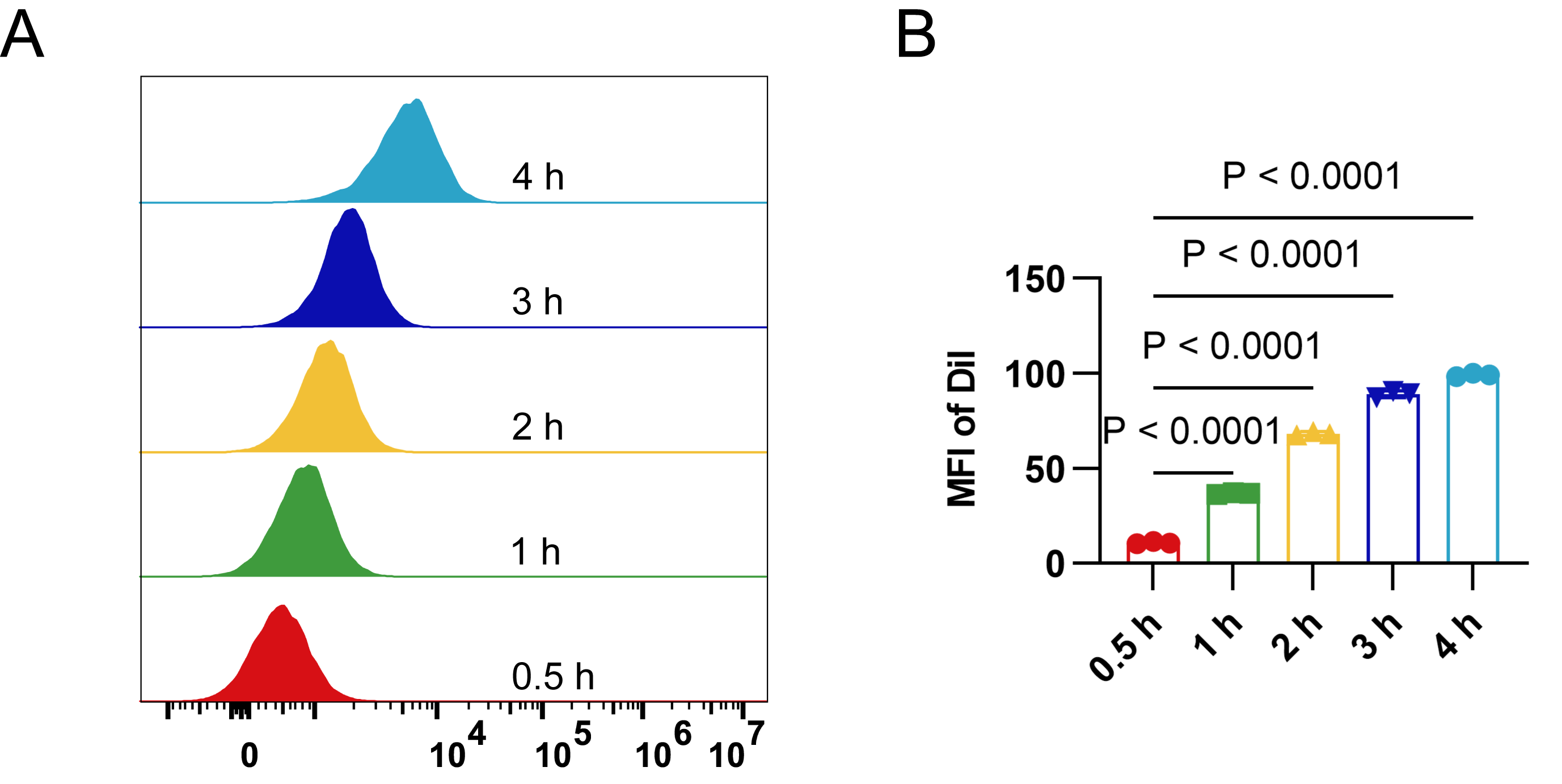


Fig. S2. (A) Intracellular uptake of PHS NPs in TC-1 cells was detected by flow cytometry, and the mean fluorescence intensity (MFI) was quantified to assess the time-dependent cellular internalization (*P< 0.0001;* B). Data are expressed as mean ± SD (n = 3). Statistical significance is indicated in the figure.


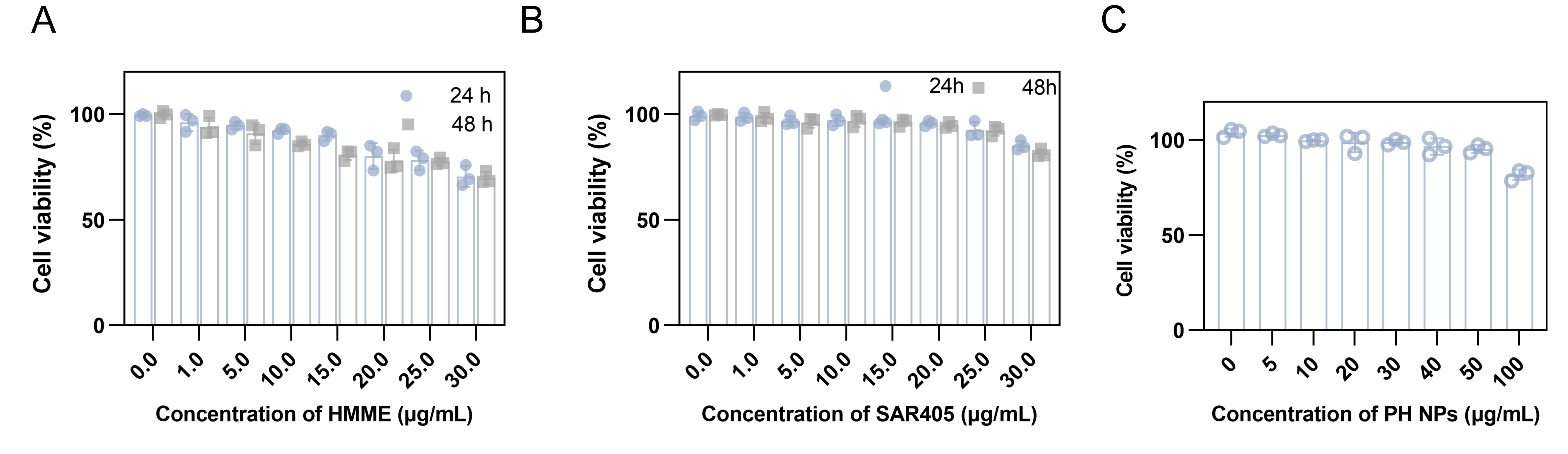


Fig. S3. Cytotoxicity evaluation of HMME, SAR405, and PH NPs in TC-1 cells. Cell viability of TC-1 cells after 24 h and 48 h incubation with varying concentrations of HMME (0–30 μg/mL) (A) and SAR405 (0–30 μg/mL) (B), measured by CCK-8 assay. (C) Dose-dependent cytotoxicity of PH NPs (0–100 μg/mL) in TC-1 cells after 24 h incubation, evaluated by CCK-8 assay.


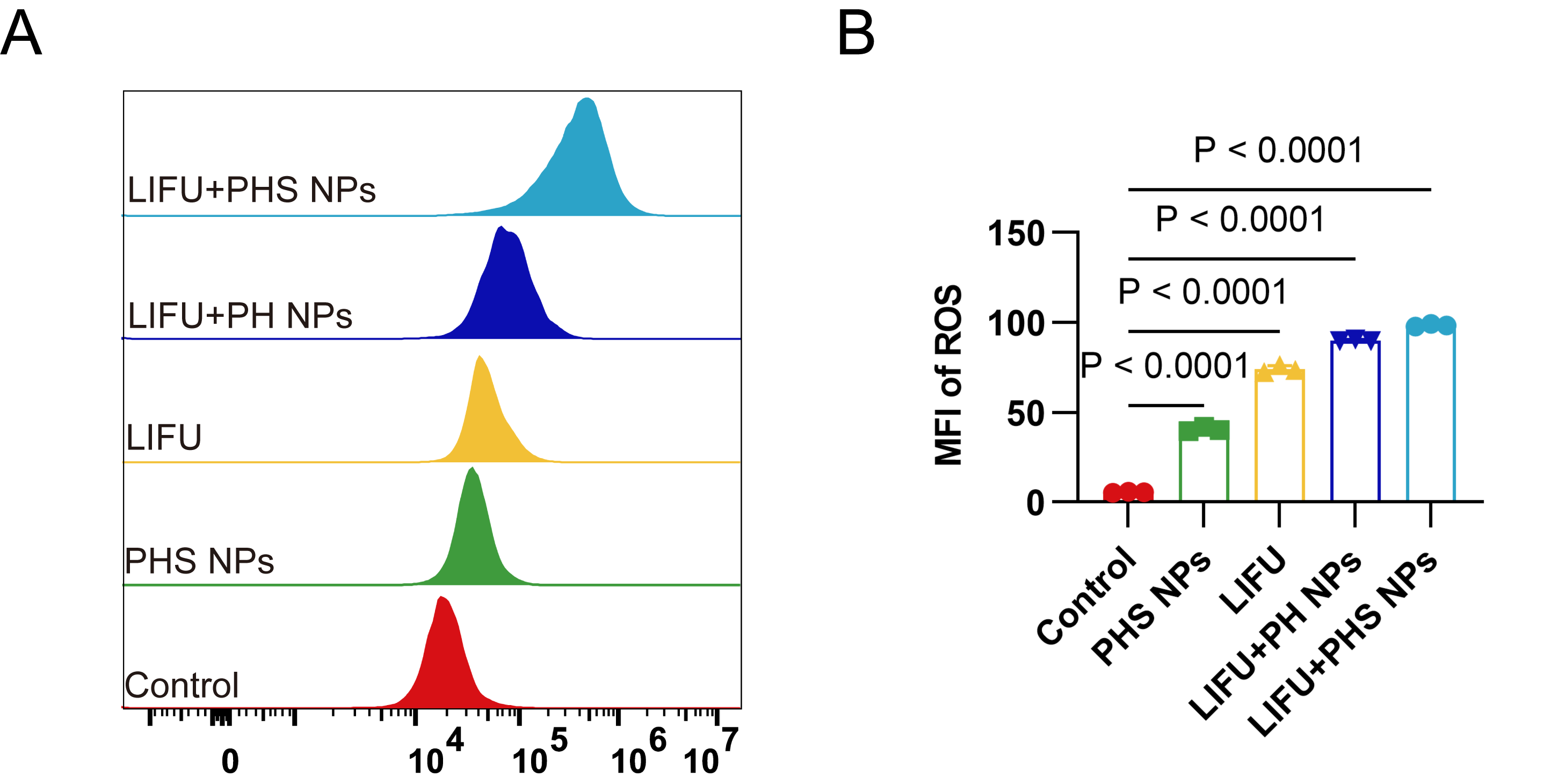


Fig. S4. (A) Representative FCM showing intracellular ROS levels in TC-1 cells detected by the DCFH-DA probe and quantification of mean fluorescence intensity (*P< 0.0001;* B). Data are expressed as mean ± SD (n = 3). Statistical significance is indicated in the figure.


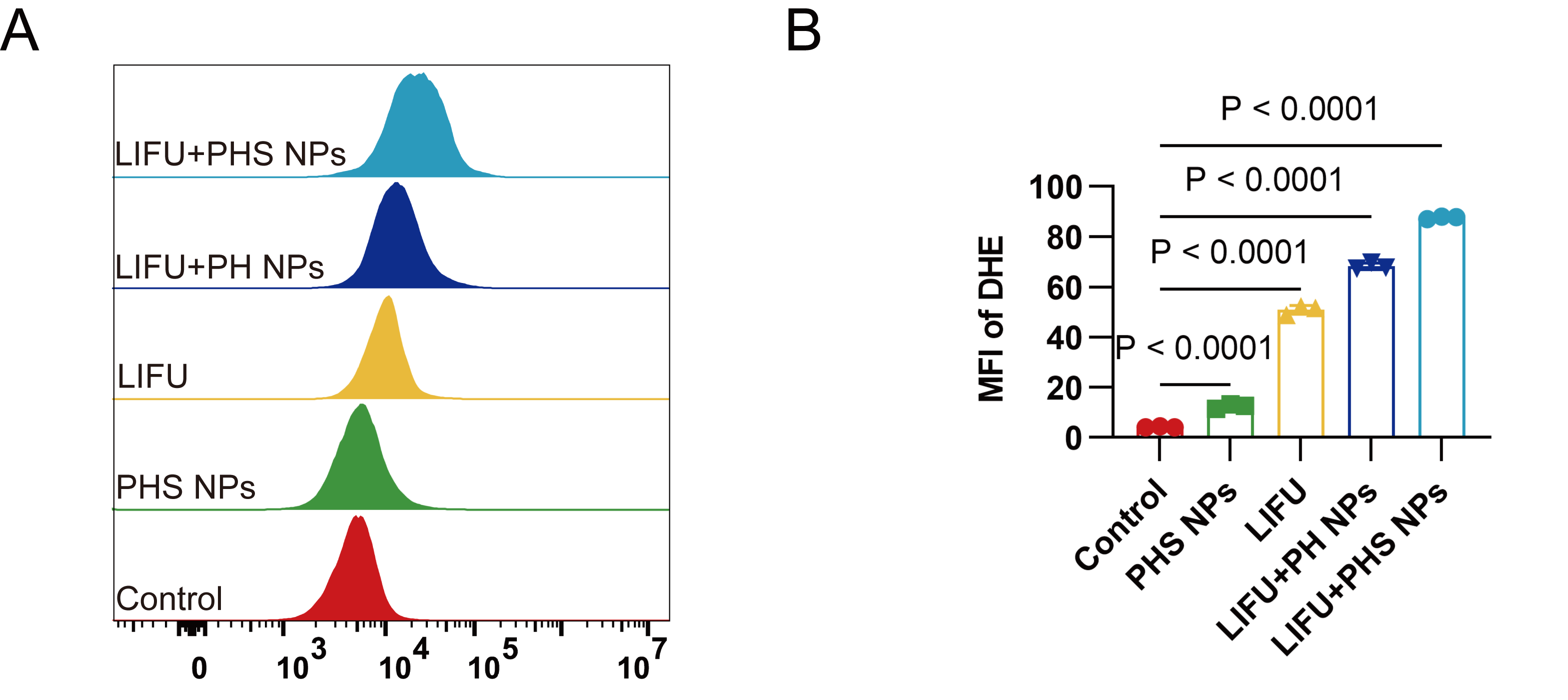


Fig. S5. (A) Representative FCM showing intracellular superoxide anion (O₂•⁻) levels in TC-1 cells detected by the DHE probe and quantification of mean fluorescence intensity (B). Data are expressed as mean ± SD (n = 3). Statistical significance is indicated in the figure.


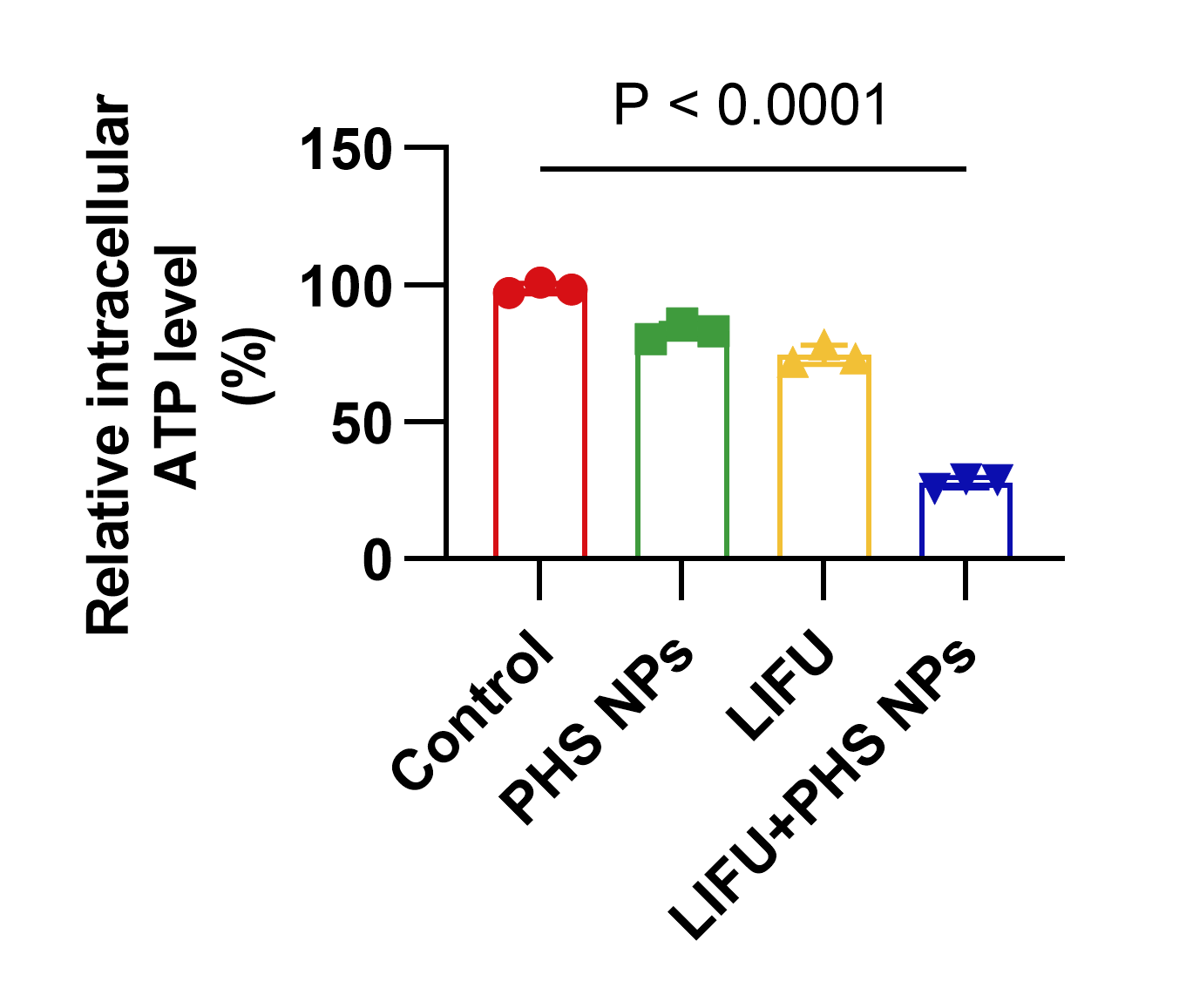


Fig. S6. Intracellular ATP levels in TC-1 cells after different treatments. Data are expressed as mean ± SD (n = 3). Statistical significance is indicated in the figure.


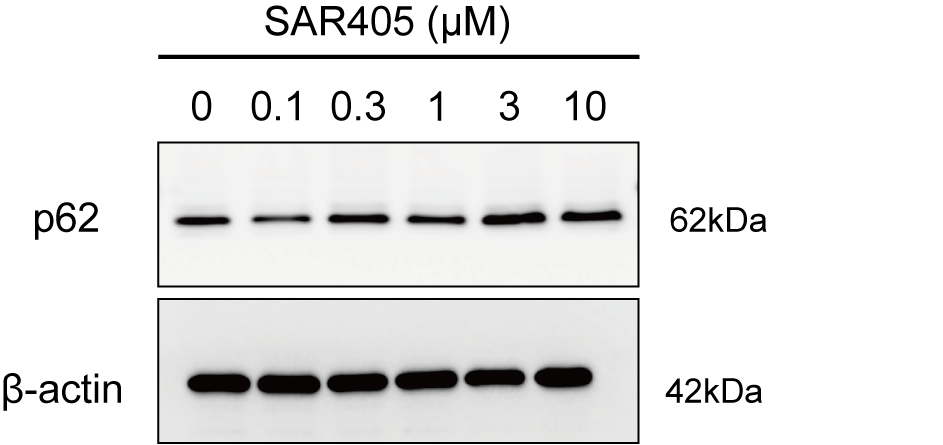


Fig. S7. Dose-dependent inhibition of autophagy by SAR405 in TC-1 cells.


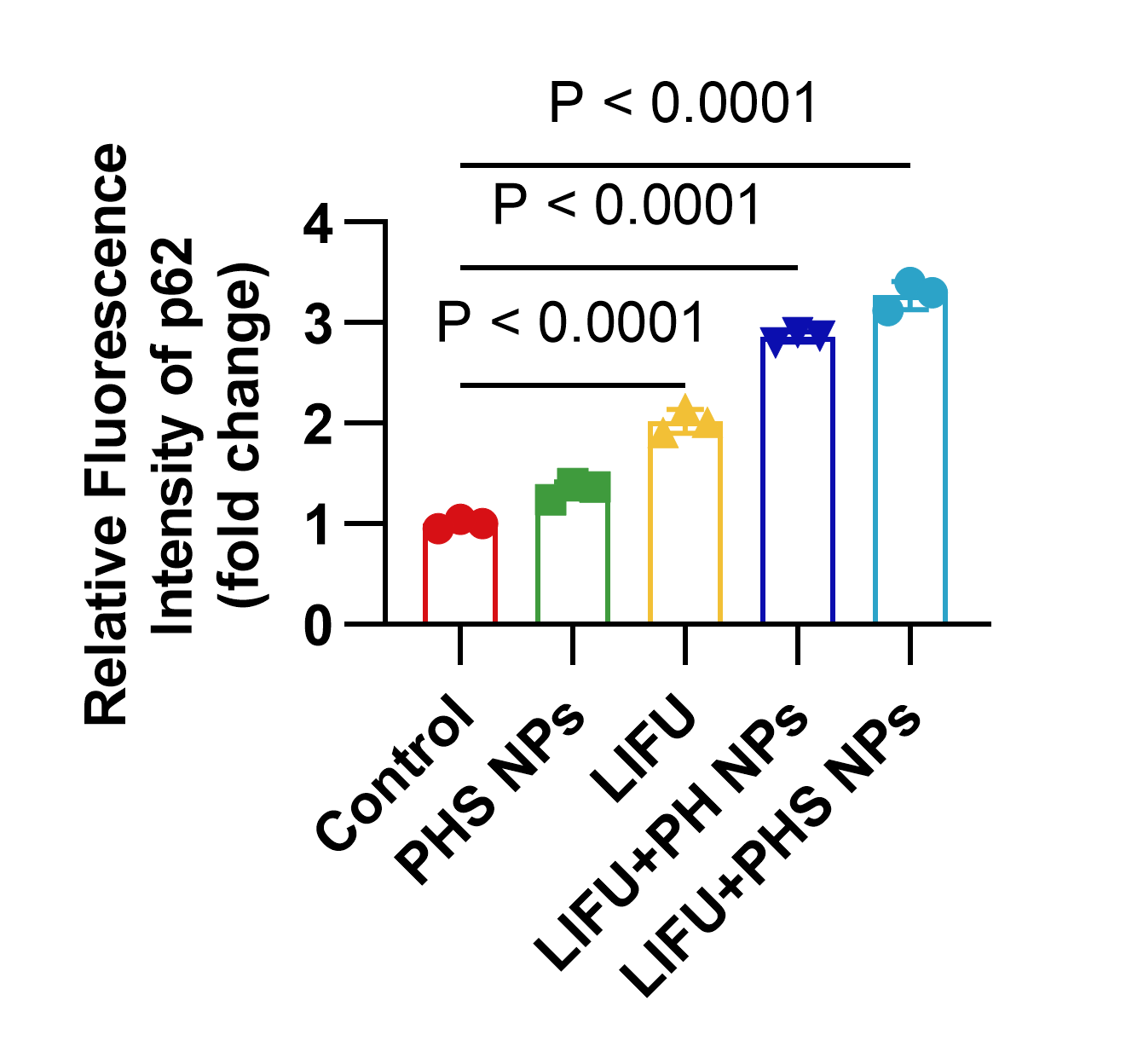


Fig. S8. Quantification of p62 fluorescence intensity. Data are expressed as mean ± SD (n = 3). Statistical significance is indicated in the figure.


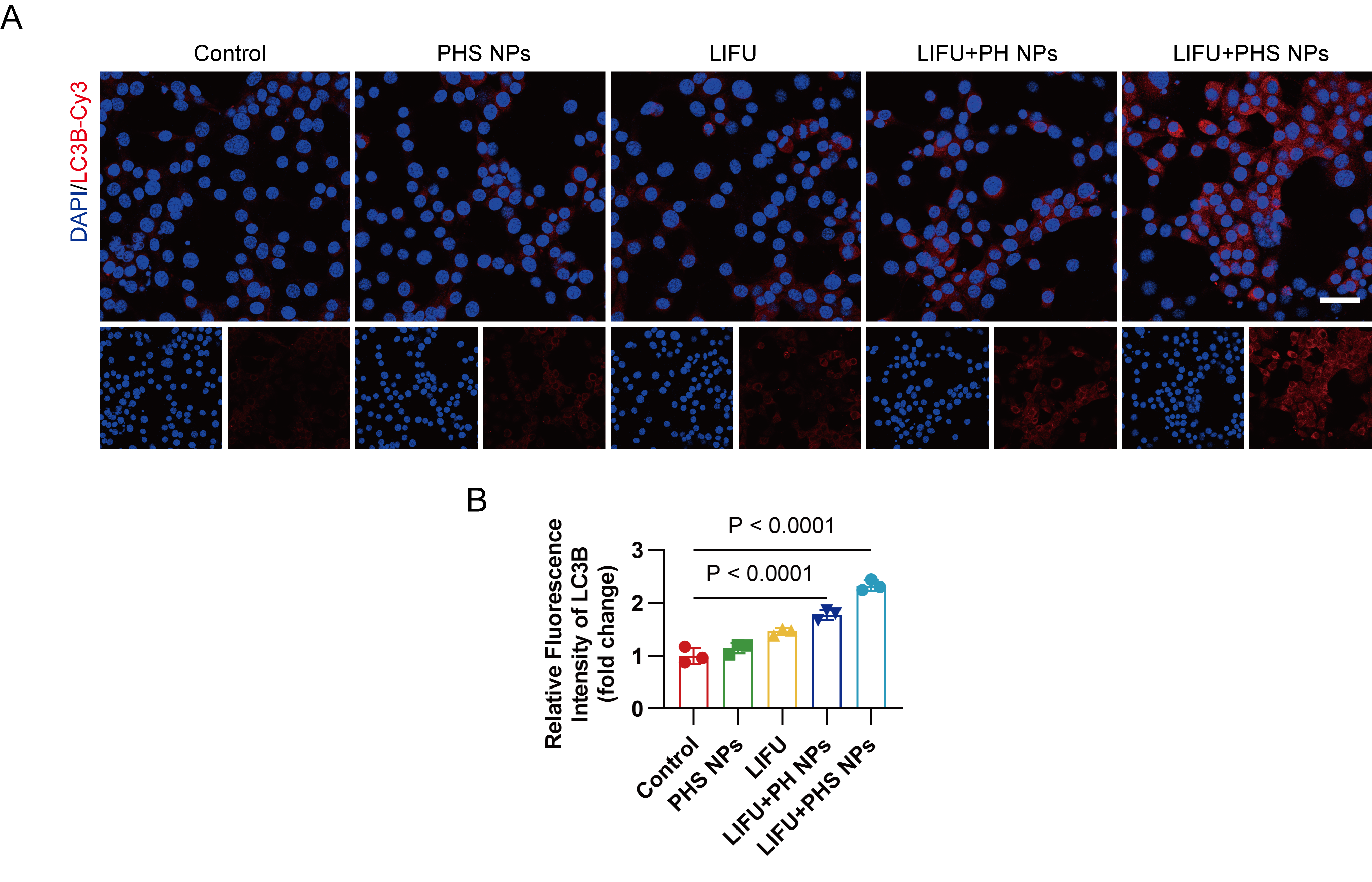


Fig. S9. (A) Representative CLSM images showing LC3 puncta formation in TC-1 cells following different treatments, and quantification of LC3 fluorescence intensity (B). Scale bar = 20 µm. Data are expressed as mean ± SD (n = 3). Statistical significance is indicated in the figure.


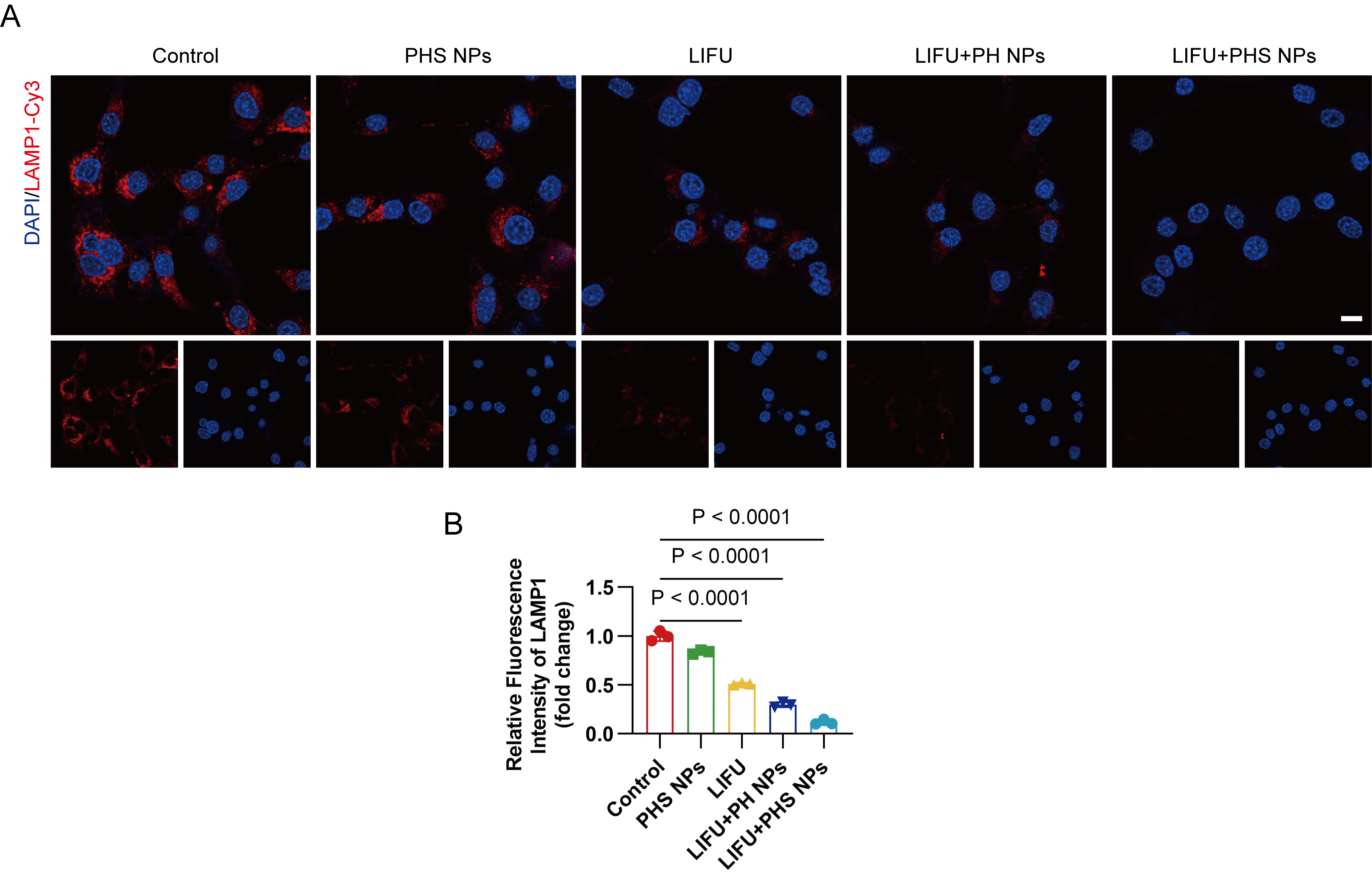


Fig. S10. (A) Representative CLSM images showing LAMP1 immunofluorescence in TC-1 cells following different treatments, and quantification of LAMP1 fluorescence intensity (B). Scale bar = 10 µm. Data are expressed as mean ± SD (n = 3). Statistical significance is indicated in the figure.


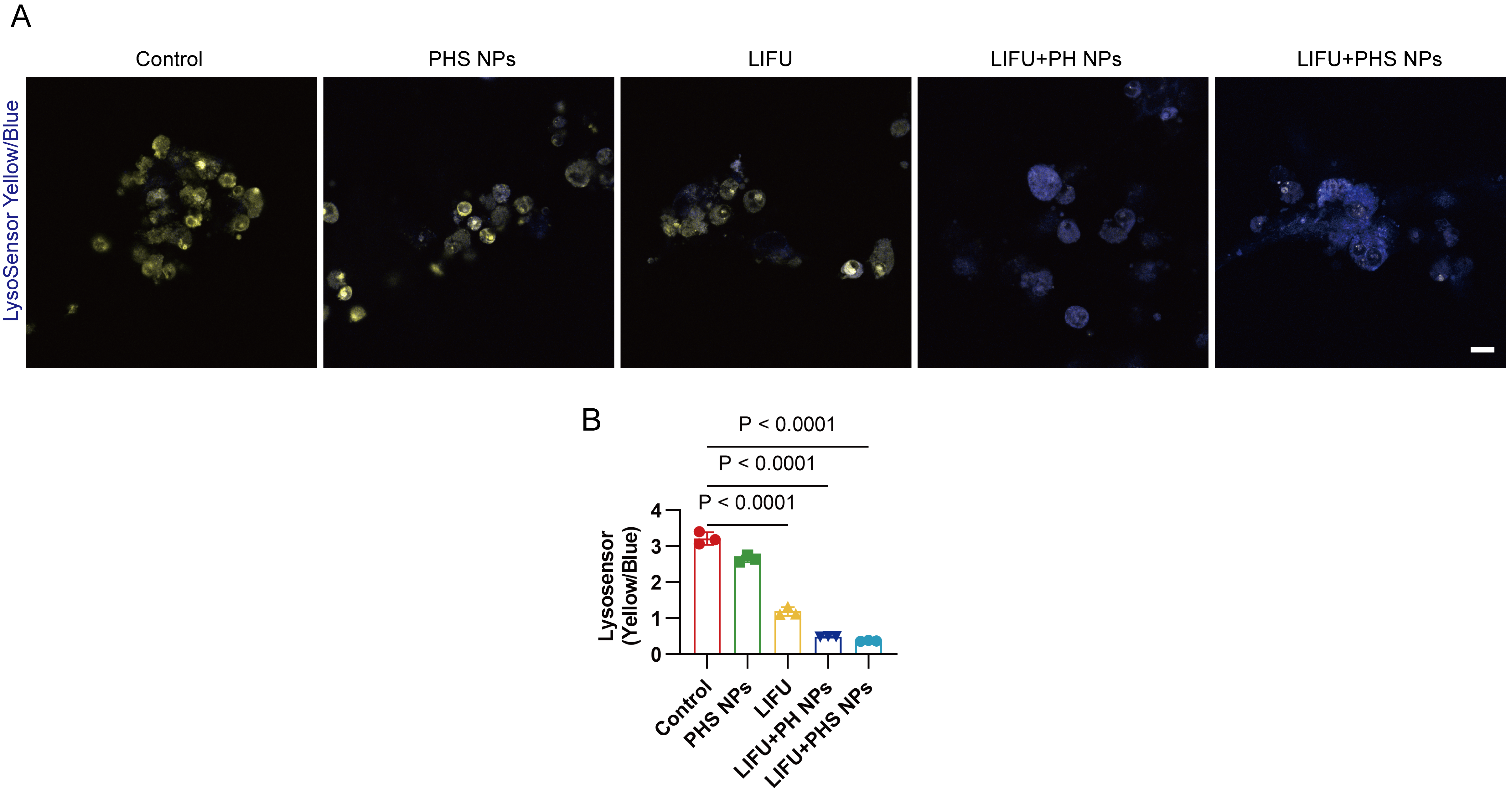


Fig. S11. (A) Representative CLSM images of TC-1 cells stained with LysoSensor Yellow/Blue following different treatments, and quantification of the Yellow/Blue fluorescence intensity ratio (B). Scale bar = 10 µm. Data are expressed as mean ± SD (n = 3). Statistical significance is indicated in the figure.


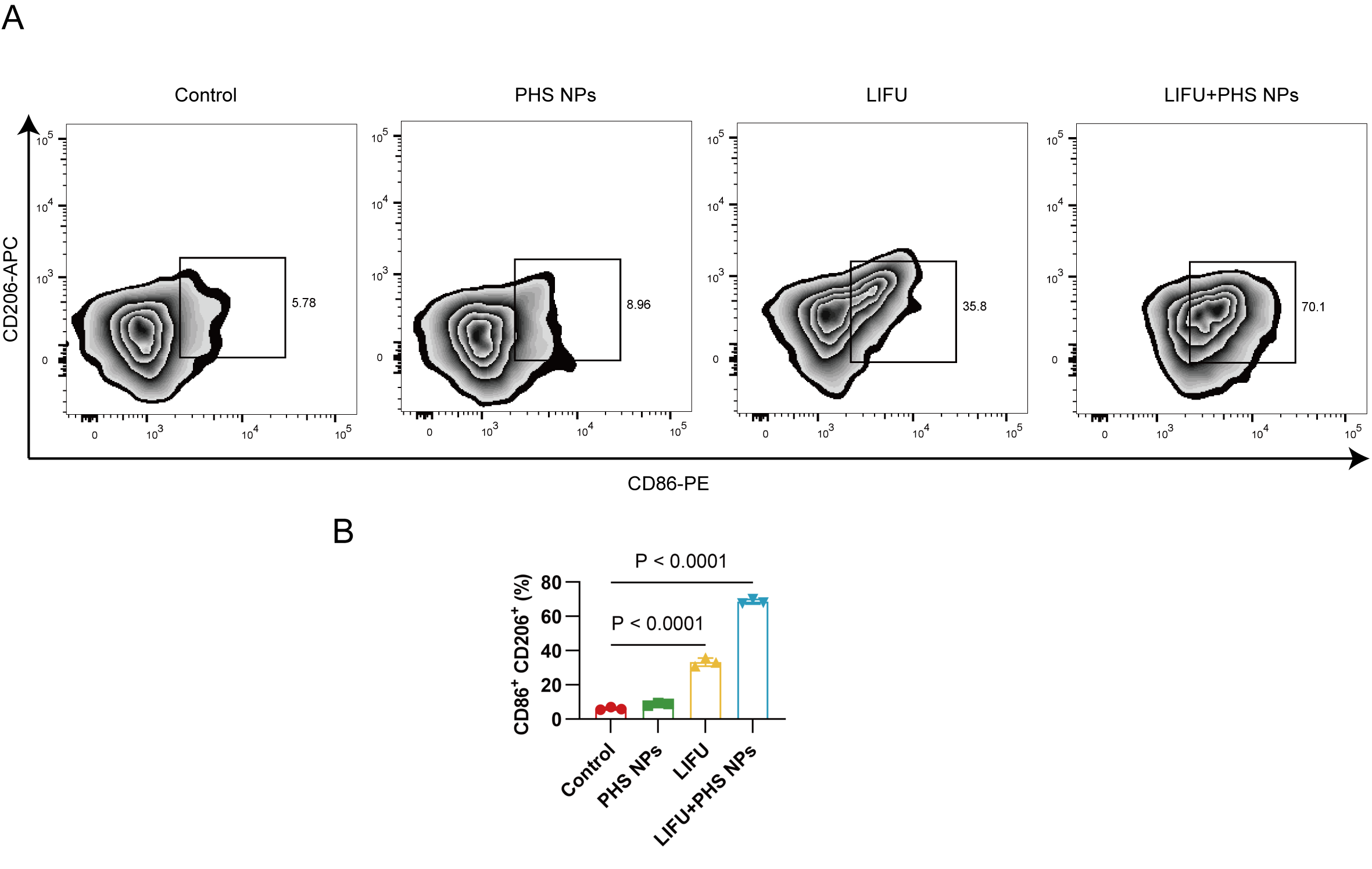


Fig. S12. (A) Representative FCM plots showing macrophage polarization in RAW264.7 cells under different treatments, and quantification of the percentage of M1-polarized (CD86⁺CD206⁻) macrophages(B). Data are expressed as mean ± SD (n = 3). Statistical significance is indicated in the figure.


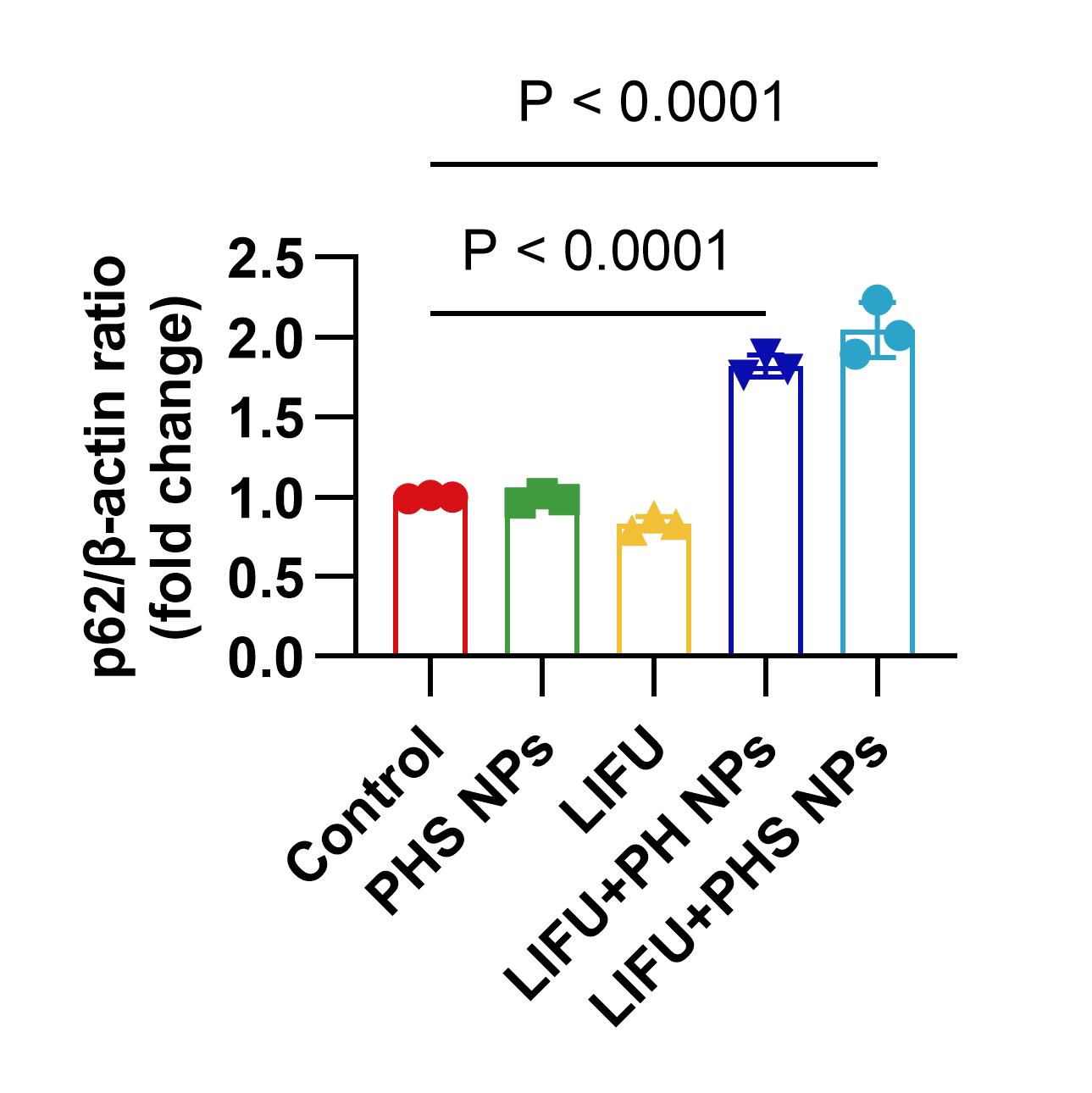


Fig. S13. Western blot analysis of autophagy-related proteins and quantification of p62/β-actin ratios. Data are shown as mean ± SD (n = 3); Statistical significance is indicated in the figure.


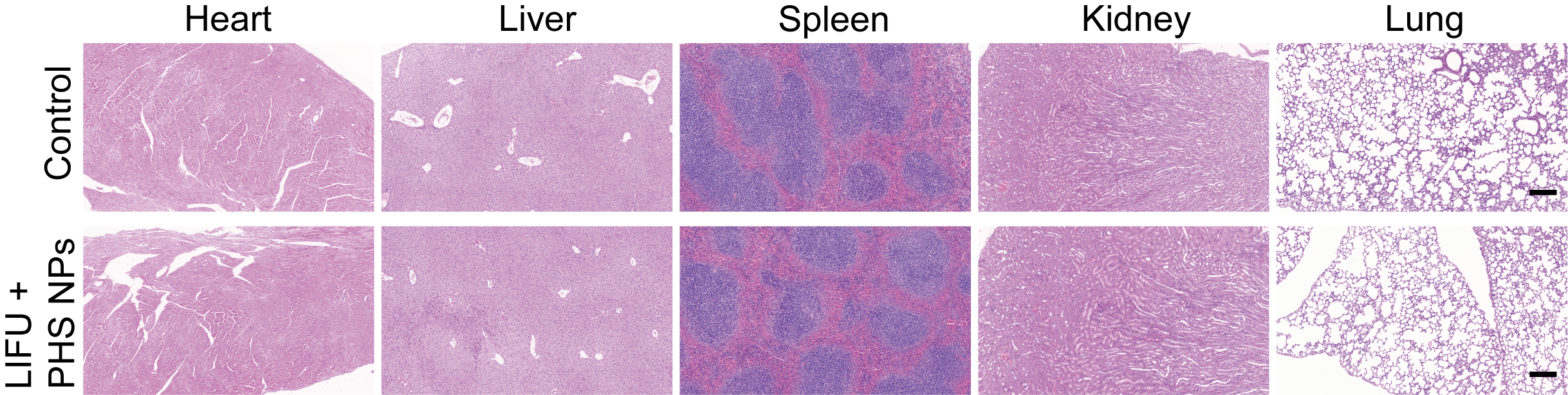


Fig. S14. Representative H&E-stained sections of major organs from mice treated with LIFU+PHS NPs. Scale bar =100 μm.
